# Comprehensive dataset on the physicochemical characteristics of agrowastes digestates from anaerobic digestion

**DOI:** 10.1016/j.dib.2025.111550

**Published:** 2025-04-08

**Authors:** Lucille Caradec, Aurélia Michaud, Mariana Moreira, Ivan Desneulin, Sylvaine Berger, Dominique Patureau, Sabine Houot, Florent Levavasseur, Antoine Savoie, Julie Jimenez

**Affiliations:** a INRAE, Institut Agro-UMR SAS, 65 rue de St-Brieuc, CS 84215, 35042 Rennes, France; b Chambre d'Agriculture de Bretagne, Rue Maurice Le Lannou, 35000 Rennes, France; c SOLAGRO, 75 voie du TOEC, 31000 Toulouse, France; d INRAE, Univ. Montpellier, LBE, 102 Avenue des étangs, 11100 Narbonne, France; e INRAE, AgroParisTech, Université Paris-Saclay, UMR ECOSYS, 91120 Palaiseau, France; f INRAE, UE PAO, 37380 Nouzilly, France

**Keywords:** Digestates, Anaerobic digestion, Characterization, organic matter, Agronomic indicators, Trace metal elements, Organic contaminants, Macronutrients

## Abstract

The established database represents one of the largest and most diverse collections of digestates compositions, with a total of 806 digestates derived from two main collections: (i) 608 digestates from French agricultural anaerobic digestion (AD) plants, which include detailed physicochemical characterization, trace metal elements and organic contaminants concentrations, and potential C and N mineralization during soil incubation in controlled conditions as agronomic indicators, and (ii) 198 digestates collected from an international literature review, with physicochemical characterization and some trace metal elements concentrations. An additional dataset of process metadata was also set-up, including feedstock compositions, operational conditions and post-treatments descriptions. A preliminary description of both collections is provided using descriptive statistics. This dataset can help to better understand the variability in digestates quality based on feedstock compositions, operational conditions and post-treatments, thereby improving their agronomic benefits.

## Graphical Abstract

**Figure:**
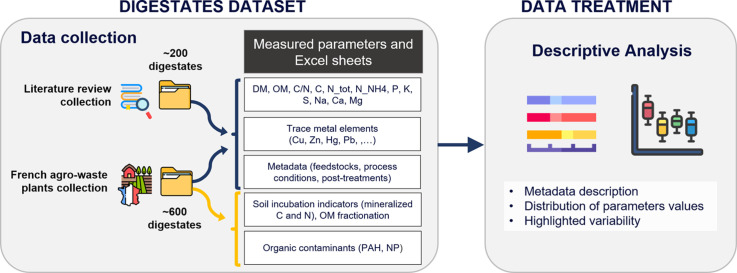

Specifications Table

**Table:** 

Subject	Waste Management and Disposal.
Specific subject area	Composition of digestates collected from anaerobic digesters located on French farms and from literature
Type of data	Table, Excel sheets
Data collection	During the national Fert-Dig French program (ADEME, 2024), two collections were created. The French digestates collection contains 608 observations from 165 anaerobic digestion (AD) plants encompassing physicochemical characteristics of digestates, as well as trace metal elements and organic contaminants concentrations and potential C and N mineralization during soil incubation in controlled conditions as agronomic indicators from 2006 to 2022. This data collection campaign has been done using INRAE internal database, data collected from French AD owners (AAMF), and technical reports from other French organisations. The second database contains 198 observations and was built based on literature review of peer-reviewed articles. The selection excludes missing information from AD process operational conditions and feedstocks ratio*.*
Data source location	INRAE internal databases, France Farmers owning anaerobic digestion plants: AAMF (Association des Agriculteurs Méthaniseurs de France), France French centralized anaerobic digestion plants (without urban feedstocks as municipal solid wastes or sewage sludge) Peer-reviewed articles : digestates obtained from agrowastes feedstocks including biowastes (without urban feedstocks as municipal solid wastes or sewage sludge) at both lab-scale and industrial scale*.*
Data accessibility	Repository name: Recherche Data Gouv, Data INRAE Data identification number: 10.57745/M1JSU5 Direct URL to data:https://doi.org/10.57745/M1JSU5 Instructions for accessing these data: use the latest version of the data containing the following extension name “_20241220”
Related research article	none

## 1 Value of the Data

• This dataset represents one of the largest collection of digestates characteristics, integrating comprehensive physicochemical properties, trace metal and organic contaminants concentrations, and key agronomic indicators, making it a unique resource for comparative analysis.
• This dataset is valuable for agricultural researchers, engineers, advisors, policymakers and farmers to enhance digestates management strategies and optimize their efficient and sustainable use as organic fertilizers.
• This dataset can be used to better comprehend the variability of digestates properties generated by a large range of feedstock types, anaerobic digestion conditions, and post-treatments.
• This dataset provides reference values that can be used to assess the impacts of digestates on soil and agroecosystems, helping to link digestates characteristics with environmental outcomes.
• This dataset supports efforts to improve nutrient recycling, assess environmental impacts, and establish quality benchmarks for digestates from different sources and process conditions.

## 2 Background

The anaerobic digestion (AD) sector in Europe is rapidly expanding, producing renewable energy (biogas) and organic fertilizers (digestates). According to 2022 data from the European Biogas Association [1], as part of the REPowerEU plan, the EU aims to produce 35 Gm^3^ of biomethane per year by 2030, a tenfold increase from current levels. In 2022, an estimated 222 to 258 Mt of digestates were generated in Europe, primarily from agricultural feedstocks (90%). With AD growth, the volume of digestates requiring management is expected to rise significantly. Understanding the agronomic quality and environmental impact of digestates on crop nutrition and soil fertility is critical for its efficient use as organic fertilizer [2]. This involves examining effects on nutrient richness, nitrogen and carbon dynamics, soil biology, and soil structure. Digestates quality varies widely due to differences in feedstocks, processes, and post-treatments, complicating the establishment of clear references. While past studies have attempted to classify digestates [3], their impact on agroecosystems has to be further investigated. A data compilation from French AD plants aims to better understand digestates variability and further optimize their management in agriculture.

## 3 Data Description

The linked repository (https://doi.org/10.57745/M1JSU5) contains two main datasets describing the characteristics of digestates produced through AD. The first collection, the French digestates collection, includes 608 samples from 165 AD plants in France, covering data collected between 2006 and 2022. The second collection, derived from literature, consists of 198 observations compiled from peer-reviewed articles. The French digestates collection is depicted in seven Excel files described in the Supplementary Material (Table S1), while the literature collection includes five Excel files detailing the digestates reported in the literature, also outlined in the Supplementary Material (Table S2). The Excel files are split into categories of parameters and information: physicochemical characterization, trace metal elements concentrations, organic matter content, characterization and potential C and N mineralization, organic contaminants concentrations and process metadata. The “Process metadata” and “Literature process metadata” datasets include key details on AD process parameters, such as temperature, hydraulic retention time, and post-treatment descriptions. They also contain information on the feedstock mass ratio associated with each produced digestate. Two glossaries, “Process glossary” and “Parameters glossary,” define all parameters and categorize various process types and feedstocks. Moreover, while the datasets are edited in French, the accompanying glossary includes the English version of all the parameters. Descriptive statistical analyses are provided to characterize the published data in both collections.

### 3.1 Metadata description

For each digestate, information about feedstock, AD processes, and post-treatments was collected. The digestates in the database originate from a highly diverse range of feedstocks and AD processes, including wet and dry AD, mesophilic and thermophilic fermentation, and varying hydraulic retention times. The use of post-treatment was also collected with specific details gathered, such as the technology (e.g., screw press or centrifuge for phase separation). Accordingly, all datasets include information about the digestate fractions: raw digestates from wet and dry AD, liquid and solid fractions obtained after solid-liquid separation, and composted solid fractions.

**Fig. 1:**
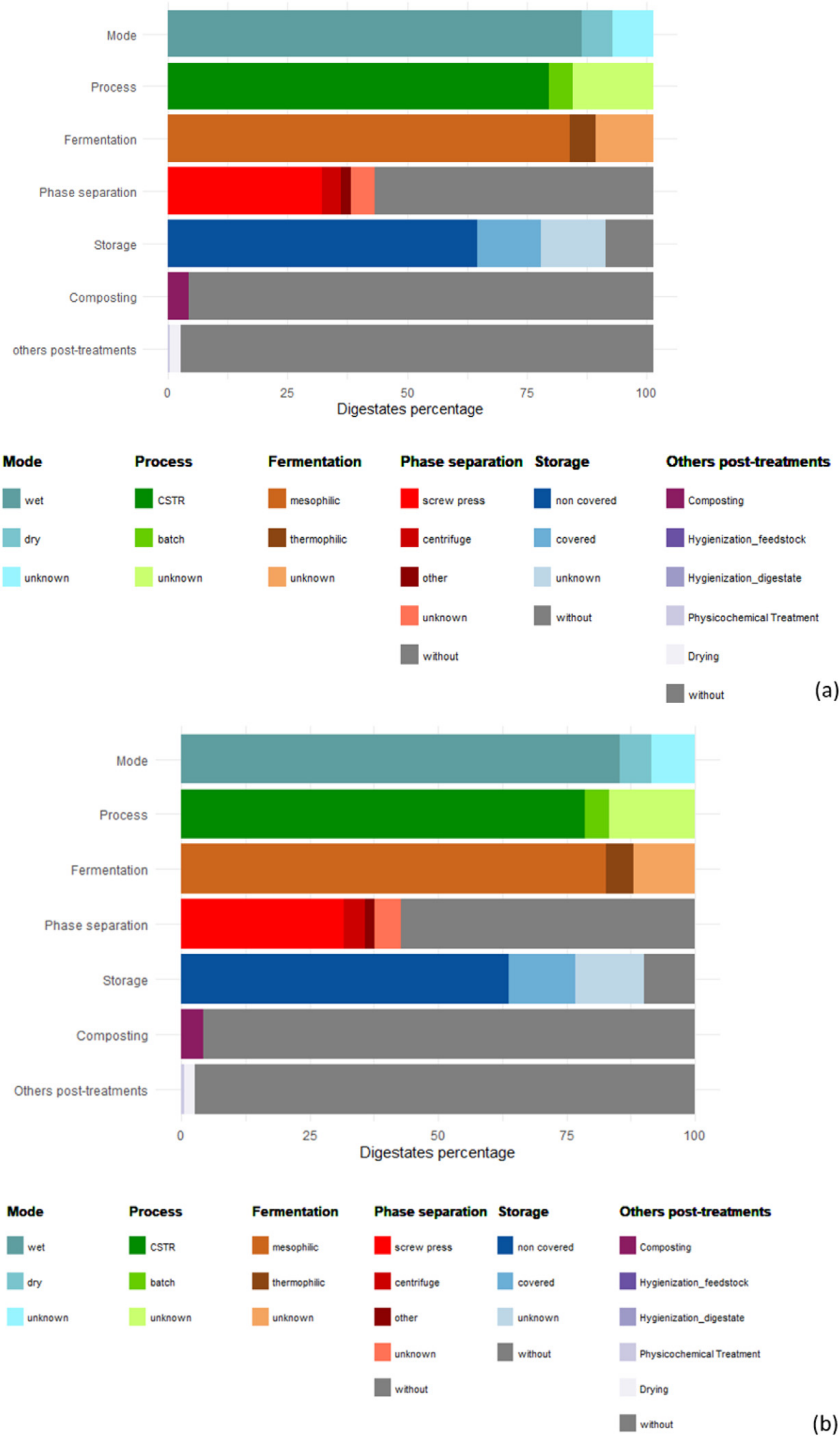
Description of anaerobic digestion process operational conditions and post-treatments involved in the French digestates collection (a) and in the literature collection (b).

To illustrate the process data, regarding the operational conditions and processes in the French agricultural AD, Fig. 1 (a) describes the data in terms of the percentage of collected observations. The most commonly found AD processes are wet (85%), continuously-fed (i.e. completely stirred tank reactor, CSTR) (79%), and mesophilic (83%) processes. Only 6% of digestates collected come from dry AD. When phase separation is applied (43% of digestates), it is most often performed using a screw press (32%) and only 4% using centrifugation. Other treatments, such as composting (4%) and drying (2%) were less frequently recorded. Collected digestates were mainly stored (90%). Regarding storage of raw and liquid digestates (75% of the reported stored digestates), since 2021, French regulations require the covering of storage tanks, except in the case of lagoons if a retention time of 80 days of digestion upstream can be justified. However, no information was recorded regarding the storage time associated with the sampling of the characterized digestates.

Regarding the literature-based dataset (Fig. 1b), digestates data from 14 countries were included: France (23%), Italy (23%), Spain (4%), Denmark (7%), Norway (5%), Germany (5%), Finland (5%), Poland (5%), Argentina (4%), Canada (4%), Belgium (4%), USA (2%), United Kingdom (1%), and Portugal (1%). The digestates in this collection mainly come from wet AD (75%), mesophilic processes (74%), and CSTR reactors (63%). However, some studies did not specify the AD process mode (24%), temperature (12%), or reactor type (35%). Fifteen percent of the observations come from thermophilic reactors, and only one digestate was reported from dry AD. Phase separation was applied to 61% of the observations, with a wider variety of techniques used compared to the French dataset. In addition to screw presses (13%) and centrifuges (4%), flocculation (8%), sedimentation (3%), vibrating screens (3%), and screw compactors (4%) were also noted. Only 3% of the reported digestates were stored, and only one was analyzed after drying. Additionally, 34% of the collected digestates were from lab-scale reactors.

**Fig. 2:**
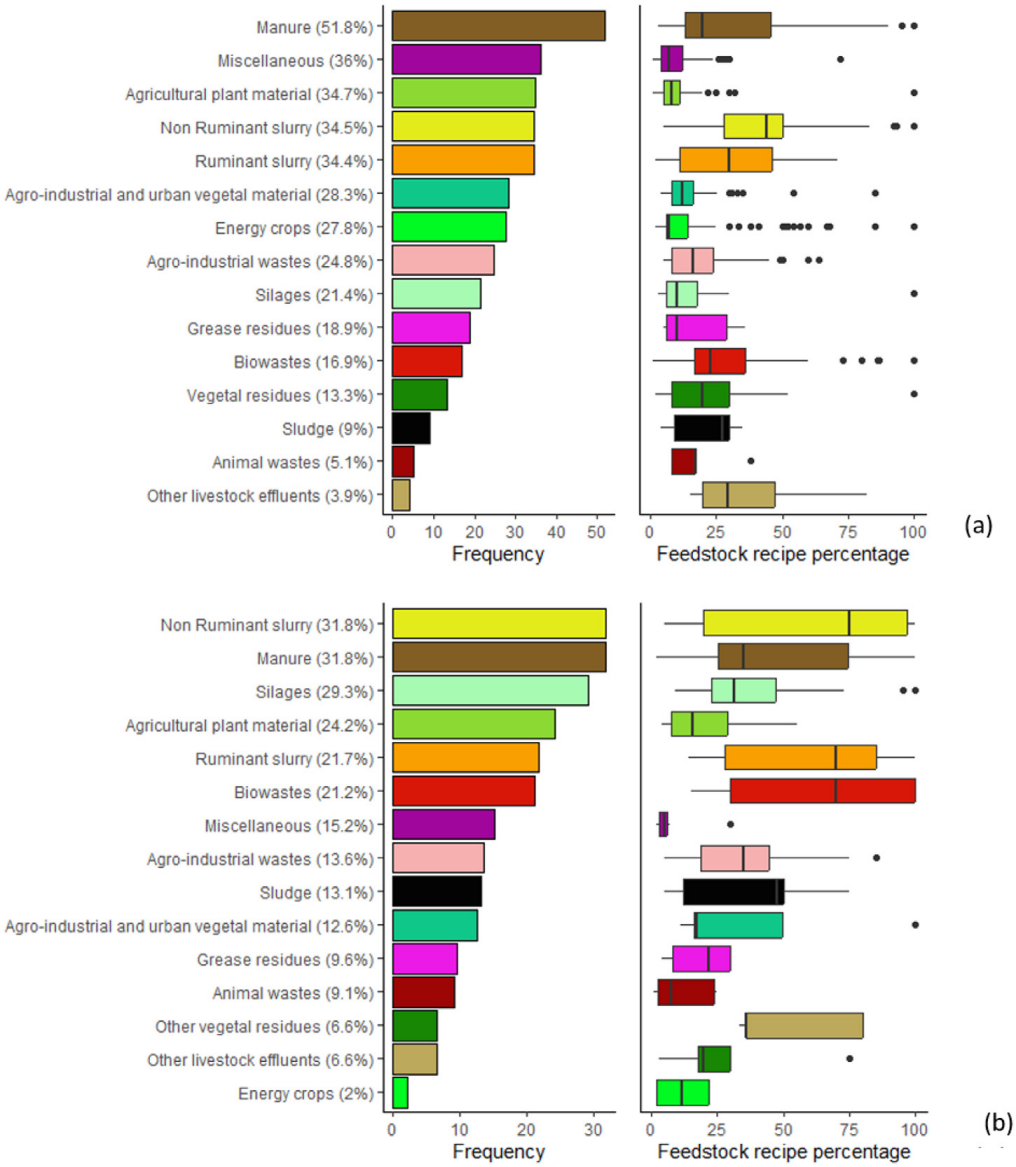
Frequency (left) of feedstocks categories and distribution in recipe percentage (right) leading to the digestates in French digestates collection (a) and in literature collection (b). The frequencies are calculated based on the number of occurrences of each feedstock category in the total dataset. Error bars represent the interquartile range, and outliers are shown as black dots.

One of the major sources of variability identified in the AD treatment chain is the feedstock type [3]. More than 96 possible feedstocks have been identified, and 15 categories were proposed to classify this wide range of agricultural waste feedstocks in the “Process metadata” dataset: manure, ruminant slurry, non-ruminant slurry, agricultural plant residues, energy crops, agro-industrial and urban vegetal residues, agro-industrial wastes, silages (other than energy crops), grease residues, biowastes, other vegetal residues, sludge, animal wastes, other livestock effluents, and miscellaneous. Fig. 2 displays the frequency of different feedstocks and shows the distribution of each feedstock category percentages in the recipe for the French (Fig. 2a) and literature (Fig. 2b) collections. In the French digestates collection, the most common feedstocks are livestock effluents, which include manure (51.8%), ruminant slurry (34.4%), and non-ruminant slurry (34.5%). Other livestock effluents are less frequently used (4%). Agricultural plant residues (34.7%) and the “Miscellaneous” category (36%), which includes various types of feedstocks, are often used as co-substrates, typically in quantities of less than 25% of the feedstock. Energy crops, agro-industrial vegetal residues, agro-industrial wastes, and silages are also commonly used (between 27% and 37%), with highly variable feedstock ratios, particularly for energy crops and agro-industrial vegetal residues. Grease residues and biowastes appear in 22% to 25% of the database, with significant feedstock ratios, sometimes exceeding 70% of the total feedstock, particularly for biowaste. In the literature-based collection, non-ruminant slurry and manure are the most reported feedstocks (31.8%). Vegetal residues, including silages and agricultural plant material, are also frequently reported, along with ruminant slurry. Biowaste is more commonly used in the literature dataset (21.2%) compared to the French dataset (16.9%). Energy crops are less frequently reported (only 2%), and agro-industrial wastes are used less frequently (13.6%) compared to the French collection (24.8%). Additionally, the feedstock mixtures in the literature dataset generally exhibit higher percentages of a single feedstock, reflecting a lower diversity in the mixtures and the fact that 34% of the digestates originate from lab-scale reactors. For example, 24 digestates in the literature collection are derived from 100% non-ruminant slurry, and 14 are from 100% biowaste.

Livestock effluents remain the dominant feedstocks, but the increasing diversity, both from farms and non-farm sources, illustrates the evolution of AD from waste treatment to a broader valorization approach.

### 3.2 Digestates characterization datasets

In both collections, 24 characteristics in common have been collected as following: Dry Matter (DM), Organic Matter (OM), Carbon (C_tot), Carbon to total Nitrogen ratio (C/N), Carbon to Organic Nitrogen ratio (C/Norg), total Nitrogen (N_total), total Ammoniacal Nitrogen (N_NH_4_), NH_4_/N ratio, total Phosphorus (P_2_O_5_) and total Potassium (K_2_O), pH, total sodium (Na2O), total calcium (CaO), total magnesium (MgO), total sulphur (SO_3_), trace elements as bore (B), copper (Cu), chromium (Cr), iron (Fe), manganese (Mn) and zinc (Zn), and fibers content (hemicellulose, cellulose, lignin).

Regarding the French digestates collection, additional parameters have been also collected as presented in supplementary material (Table S1) including: 11 additional trace metal elements, 3 indicators describing the stability and stability of residual C and N (Indicator of the potential Residual Organic Carbon, IROC, proposed by [4], mineralized C, C91 and N, N91 after 91 days of soil incubation), 18 parameters related to organic matter and nitrogen bioaccessibility fractionation associated with 35 indicators coming from to 3D fluorescence spectroscopy as proposed by [5] and 23 organic contaminants (polycyclic aromatic hydrocarbons, nonylphenols and some pharmaceuticals).

Table 1, Table 2 provide a statistical overview of the collected values for the French digestates collection and the literature collection respectively. Depending on the category of parameters, the number of observations is variable. Global physicochemical parameters are the most frequently referenced compared to trace metal elements, soil incubation-related indicators or organic contaminants. Indeed, from the 608 digestates collected in the French digestates collection, classical physicochemical parameters such as DM, OM, nitrogen forms, K_2_O and P_2_O_5_ concentrations are thoroughly reported, with missing data ranging from 3% to 15% whereas Na_2_O, CaO, MgO and SO_3_ concentrations exhibit between 52% and 83% of missing data, trace metal elements are reported with 72% of missing data in average, followed by mineralized C91 and N91 indicators (82% and 87% of missing data) and the organic contaminants (more than 96%). Similar trend is observed for the 198 digestates collected in the Literature collection, with missing data ranging from 8% to 63% for the global physicochemical analysis and above 83% for trace metal elements and Na_2_O, CaO, MgO and SO_3_ parameters.

**Table 1:** Statistics overview of the collected values for the main parameters physicochemical parameters, trace metal elements and some organic contaminants for the French collection of digestates.

Category	Parameter	Median	1st quartile	3rd quartile	Mean	%CV	%Missing data
Global physicochemical parameters (g .kg DM^-1^) n = 608	DM (%RM)	8.39	5.70	17.55	13.21	99%	3%
	OM	677.00	618.10	739.56	674.95	16%	9%
	C_tot	356.99	318.50	391.49	354.89	18%	8%
	N_tot	62.16	34.76	84.52	69.76	76%	7%
	N_NH_4_	28.02	11.43	50.21	37.69	112%	12%
	N_org	30.74	22.01	39.60	33.75	62%	15%
	K_2_O	54.59	32.93	73.68	60.94	74%	22%
	P_2_O_5_	28.33	19.95	41.00	33.52	61%	18%
	pH	8.10	7.90	8.52	8.17	14%	41%
	C/N	5.79	4.00	10.23	8.10	75%	13%
	C/Norg	11.37	8.68	16.42	14.74	101%	21%
	NH_4_/Ntot	0.47	0.30	0.60	0.45	47%	15%
	Na_2_O	7.50	3.83	17.20	13.73	133%	74%
	CaO	35.46	28.70	46.70	40.44	54%	52%
	MgO	10.05	6.84	13.00	11.02	72%	52%
	SO_3_	12.50	7.05	17.20	13.23	54%	83%
Trace metal elements (g .kg DM^-1^) n = 155	Ag	0.12	0.09	0.34	0.30	135%	79%
	Al	3521.67	2180.00	5736.25	4422.60	70%	78%
	As	1.64	1.20	2.33	2.54	143%	79%
	B	33.00	24.10	43.80	35.83	42%	77%
	Cd	0.43	0.24	0.57	0.60	166%	77%
	Co	2.56	1.62	4.48	6.45	373%	79%
	Cr	7.55	1.80	16.29	11.03	117%	77%
	Cu	78.45	44.33	149.25	103.20	74%	77%
	Fe	6170.00	2820.00	10676.20	10323.89	112%	75%
	Hg	0.12	0.05	0.50	1.17	248%	79%
	Mn	324.13	230.50	443.91	348.85	53%	75%
	Mo	3.50	2.69	5.20	5.02	142%	79%
	Ni	9.81	6.94	14.49	11.53	59%	77%
	Pb	4.86	3.40	8.63	8.85	138%	77%
	Se	2.26	1.61	8.08	8.37	173%	79%
	Ti	130.43	0.04	426.11	247.01	116%	79%
	Zn	276.50	187.25	546.73	438.73	90%	77%
Potential C and N mineralization(% C or %N_org) n = 535	C91	31.13	22.61	39.93	33.13	43%	82%
	N91	16.00	2.68	30.75	18.42	140%	87%
Polycyclic aromatic hydrocarbons (µ .kg DM^-1^) n = 45	Flt	38.00	23.88	122.00	144.20	174%	96%
	BbF	54.00	14.25	212.50	129.13	122%	97%
	BaP	40.00	8.75	172.75	90.44	134%	96%

Number of digestates in each category(n), Coefficient of variation (CV), number of digestates (N), raw matter (RM), dry matter (DM), organic matter concentration (OM), total carbon concentration (C_tot), total nitrogen concentration (N_tot), ammonia concentration (N_NH_4_), total potassium (K_2_O), total phosphorous concentration (P_2_O_5_), carbon to total nitrogen ratio (C/N), carbon to organic nitrogen ratio (C/Norg), ammonia on total nitrogen ratio (NH_4_/Ntot), total sodium concentration (Na_2_O), total calcium concentration (CaO), total magnesium concentration (MgO), total sulphur concentration (SO_3_), silver (Ag), aluminium (Al), arsenic (As), bore (B), cadmium (Cd), cobalt (Co), chromium (Cr), copper (Co), iron (Fe), mercury (Hg), manganese (Mn), molybdate (Mo), nickel (Ni), lead (Pb), selenium (Se), thallium (Tl), zinc (Zn), Mineralized organic carbon (C91) and organic nitrogen (N91) in soil after 91 days, Fluoranthene (Flt), benzo(b)fluoranthene (BbF) and benzo(a)pyrene (BaP).

**Table 2:** Statistics overview of the collected values for the main parameters physicochemical parameters and some trace metal elements on the literature collection.

Category	Parameter	Median	1st quartile	3rd quartile	Mean	%CV	%Missing data
Global physicochemical parameters (g .kg DM^-1^) n = 191	DM (%RM)	4.90	2.50	12.45	10.08	122%	8%
	OM	683.00	617.00	765.15	678.16	20%	38%
	C_tot	341.50	308.50	382.58	339.08	20%	38%
	N_tot	58.00	33.20	108.85	79.86	85%	34%
	N_NH_4_	28.60	15.19	66.06	49.15	112%	49%
	N_org	31.95	20.76	40.90	36.21	75%	56%
	K_2_O	68.08	33.32	90.16	63.93	62%	79%
	P_2_O_5_	26.11	21.30	50.84	38.80	72%	83%
	pH	8.10	7.60	8.30	8.03	8%	42%
	C/N	6.49	3.37	10.85	8.54	83%	42%
	C/Norg	11.50	7.99	17.09	15.86	99%	63%
	NH_4_/Ntot	0.43	0.30	0.62	0.47	45%	56%
	Na_2_O	28.96	16.57	40.10	28.16	53%	93%
	CaO	56.30	33.36	63.11	51.54	46%	93%
	MgO	174.80	75.86	256.14	180.95	65%	93%
	SO_3_	24.00	14.19	25.88	25.42	70%	91%

trace metal elements concentrations (g .kg DM^-1^) n = 42	B	78.75	68.73	104.88	89.39	47%	94%
	Cu	208.30	80.08	661.60	486.76	171%	89%
	Cr	34.65	22.48	125.83	100.67	121%	95%
	Fe	2350.00	2100.00	3450.00	2957.14	62%	93%
	Mn	331.45	232.65	521.90	396.34	64%	93%
	Zn	315.60	43.15	1159.55	856.40	150%	88%

Number of digestates in each category(n), Coefficient of variation (CV), number of digestates (N), raw matter (RM), dry matter (DM), organic matter concentration (OM), total carbon concentration (C_tot), total nitrogen concentration (N_tot), ammonia concentration (N_NH_4_), total potassium (K_2_O), total phosphorous concentration (P_2_O_5_), carbon to total nitrogen ratio (C/N), carbon to organic nitrogen ratio (C/Norg), ammonia on total nitrogen ratio (NH_4_/Ntot), total sodium concentration (Na_2_O), total calcium concentration (CaO), total magnesium concentration (MgO), total sulphur concentration (SO_3_), bore (B), chromium (Cr), copper (Cu), iron (Fe), manganese (Mn), zinc (Zn).

From both Table 1, Table 2, digestates exhibit a large variability in all the collected parameters, as indicated by the coefficient of variation (CV) and the broad ranges described by the first and third quartiles. The global physicochemical parameters present CV ranging from 54% to 133% except for pH, OM and C_tot which show lower values (14%, 16% and 18% respectively) in the French digestate collection. Similar trends are observed in the literature collection, with CV going from 45% to 122% for physicochemical parameters, again with lower values for pH, OM and C_tot (8%, 20% and 20% respectively). Regarding the French digestates collection, high variability is also noted for other categories of characterization parameters, such as trace metal elements (CV above 42%), mineralized carbon and nitrogen indicators C91 and N91 (CV of 43% and 140% respectively) and organic contaminants (CV above 122%).

**Fig. 3:**
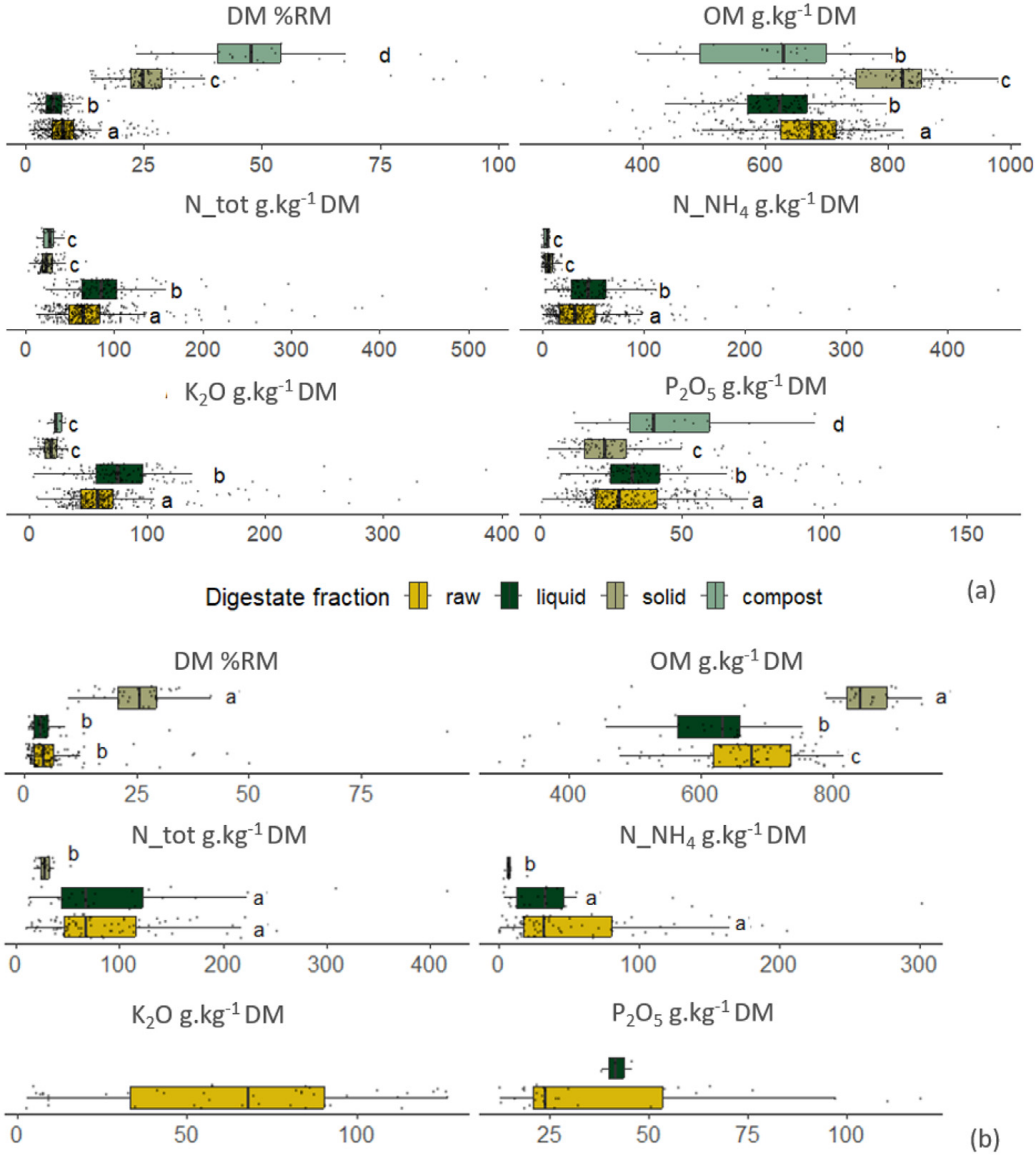
Boxplots obtained from “Physicochemical characterization” of the collected digestates dataset from (a) French digestates and (b) literature collection.

To further describe the variability of digestates, an *a priori* classification based on post-treatments that generate various fractions (i.e., raw, liquid, solid through phase separation, and compost from the solid digestates) was proposed. These products are considered with very different spreading logistics (spreading schedule, equipment, ...) because of their structure and quality. Based on the data collected in the French digestates collection, 57% of the observations are raw digestates, 21% are liquid digestates obtained after phase separation, 18% are solid digestates after phase separation, and 4% are composted solid fractions. In the literature collection, 61% of the digestates are raw, 20% are liquid fractions, and 19% are solid fractions. Fig. 3a and b, along with Tables S3 and S7 in the Supplementary Material, illustrate the physicochemical composition of the collected digestates for both the French and literature collections. Despite this classification by fraction, the digestates still show significant variability in some parameters, as shown by the boxplots. Nutrients, such as nitrogen forms (N_tot, N_NH_4_) and K₂O, are predominantly found in the raw and liquid fractions, while less soluble elements like DM and OM concentrate in the solid fraction and compost, as a consequence of phases separation process. Regarding P₂O₅, the highest concentrations are observed in compost, followed by liquid, raw, and solid digestates. Statistical analyses confirm significant differences between fractions for DM, OM, and P₂O₅, as shown in Fig. 3a. N_tot, N_NH_4_ and K_2_O content are not statistically different between composts and solid digestates. Although raw and liquid digestates from the French collection have similar value ranges, the Wilcoxon test indicates that they are statistically different for all the parameters. In contrast, values for DM, N_tot, and N_NH4 are not statistically different in the Literature collection. Overall, the physicochemical characterization of digestates from the literature and French agricultural collections aligns well in terms of concentration ranges across fractions.

*Different letters indicate when significant differences appeared between groups based on Tukey's post-hoc test for ANOVA or Dunn's test for Kruskal-Wallis (p < 0.05). (RM: raw matter, DM: dry matter content in percentage of RM, OM: organic matter concentration in g.kg^-1^ DM, N_tot: total nitrogen concentration g.kg^-1^ DM, N_NH_4_: ammonia concentration in g.kg^-1^ DM, K_2_O_total: total potassium concentration in g.kg^-1^ DM, P_2_O_5__total: total phosphorous concentration in g.kg^-1^ DM)*

**Fig. 4:**
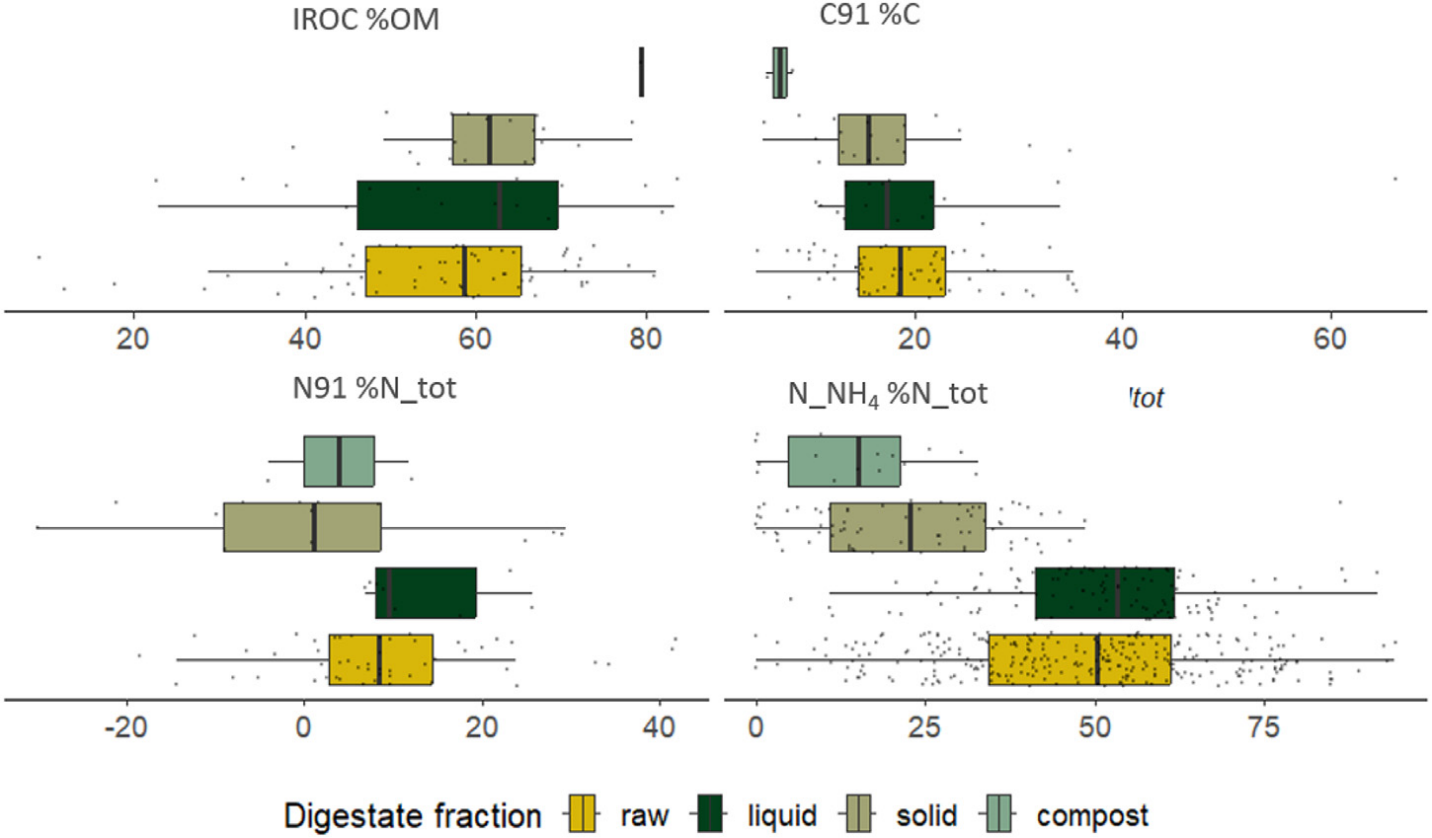
Boxplots obtained from “Soil mineralized organic matter and fractionation” of the collected digestates dataset in the French digestates collection. *Different letters indicate when significant differences appeared between groups based on Tukey's post-hoc test for ANOVA or Dunn's test for Kruskal-Wallis (p < 0.05). (IROC: Indicator of potential Residual Organic Carbon in percentage of organic matter, C91: mineralized organic carbon after 91 days of soil incubation in percentage of organic carbon, N91: mineralized organic carbon after 91 days of soil incubation in percentage of total nitrogen, N_NH_4_: ammonia content in percentage of total nitrogen).*

Regarding the indicators obtained from agronomic characterization through soil incubations tests under controlled conditions and organic matter fractionation collected in the French digestates collection, the 91-day mineralizable carbon (C91) and organic nitrogen (N91) as well as the stability indicator IROC proposed by [4] are displayed in the boxplots from Fig. 4 and in Supplementary data (Table S6). High variability is observed in the digestate fractions (raw, liquid, and solid) for IROC and C91, indicators associated with their amendment potential. Composts have low variability due to a limited data set but demonstrate high OM stability and amendment potential. However, the significant variability in values prevents the differentiation of solid digestates from raw and liquid fractions. Similar variability is observed for N91 and the proportion of ammoniacal nitrogen in total nitrogen, where N91 is higher in raw and liquid digestates compared to solid and composts. Although composts show lower variability, raw and liquid fractions maintain high levels of N_NH_4_.

Other organic matter characterization parameters, such as OM bioaccessibility fractionation [5], are detailed in the Supplementary material (Table S6) showing also high variability. When combined with 3D fluorescence data, the OM nature of digestates can be analyzed, providing insights into biodegradation potential [5].

Despite the limited dataset, statistics on trace metal elements content and organic contaminants collected in the French digestates collection are provided in the Supplementary Material (Table S4 and Table S5). Significant variability is observed for Cu, Zn, and Cr. Since trace metals are conserved during the AD process, digestates coming from feedstocks with high levels of metals should be monitored and compared to regulatory thresholds. Globally, values are lower than European Regulation UE 1009/2019 thresholds for Pb, As and Ni, with some outliers regarding Zn, Cd, Cu and Hg as shown in Supplementary Material (Fig. S1). Regarding organic contaminants concentrations, values are very low compared to European regulations thresholds (Supplementary Material, Table S5).

Despite some trends in differentiation by fraction classification, the high variability of some digestates characteristics requires further investigation. The interaction between feedstock types, post-treatment methods, and AD process conditions needs to be explored further using these datasets, in line with the perspectives of previous studies [3], to better understand and explain the variability of digestates.

## 4 Experimental Design, Materials and Methods

Literature collection was obtained through an exhaustive literature review on published papers from 2008 to 2020. 36 peer-reviewed scientific articles were selected (Table S9, Supplementary data). The selection of the digestates described on the literature was done when enough metadata was provided (feedstock, AD operational conditions, post-treatment description) and was focused on physicochemical parameters. Both lab-scale and industrial-scale were considered. The literature review was focused on agrowaste feedstocks, including biowaste whereas sewage sludge and municipal solid wastes were excluded.

To establish a large digestates database, unpublished data provided by INRAE, the French Association of Agricultural Anaerobic Digestion (AD farmers owners), and the French Chambers of Agriculture were collected from 2006 to 2022. As for literature review, the digestates characteristics coming from urban wastes (sewage sludge and municipal solid wastes) were not collected.

The several analytical methods associated with physicochemical characterization and trace metal elements datasets are described in Table 3. Each digestate was analysed, with the number of replicates varying across the measured parameters from the numerous sources (from 2 to 3 replicates on the physicochemical parameters). Statistical analyses were performed on the average values of the available replicates for each sample and each measured variable.

**Table 3:** Analytical methods associated to physicochemical characterization and trace metal elements datasets from the French agricultural digestates collection.

Parameters	Analytical methods
Total and organic Carbon (C_total and C_org)	Dry combustion NF ISO 10694 / Dumas method /Dumas with carbonates correction / Combustion and infrared detection / Sulfochromic oxydation
Dry matter (DM)	105°C, NF EN 13040 / Drying 103°C ± 5°C / NFU 44110
Organic matter (OM)	Combustion 550°C / Calcination
Ammonium (N_NH_4_)	N Direct Distillation / KCl extraction on fresh matter / NF EN 13652 on fresh matter / KCl extraction / Ionic Chromatography / Colorimetry and H_2_SO_4_ titration
Organic nitrogen (N_org)	Calculation N_total – N_NH4 Combustion and infrared detection / Combustion at 550°C
Total nitrogen (N_tot)	Kjeldahl / Modified Kjeldahl /Colorimetry
Total potassium (K_2_O_total)	Regal water extraction /Extraction by acid digestion /Combustion at 550°C Inductively coupled plasma mass spectrometry / Mineralization on dry matter NF EN 13346, NF EN ISO 11885
Total phosphorous (P_2_O_5__total)	Regal water extraction /HNO_3_ / H_2_O_2_ extraction/ Combustion at 550°C /Hydrofluoric acid extraction /Ammonium acetate extraction/Persulfate extraction Inductively coupled plasma mass spectrometry/Colorimetry Mineralization on dry matter NF EN 13346, NF EN ISO 11885
Total magnesium, total calcium, total sodium, total sulfur (MgO, CaO, Na_2_O, SO_3_)	Regal water extraction/Hydrofluoric acid extraction Inductively coupled plasma mass spectrometry
Trace metal elements (Ag, Al, As, B, Cd, Co, Cr, Cu, Fe, Hg, Mn, Mo, Ni, Pb, Se, Tl, Zn)	Regal water extraction/Hydrofluoric acid extraction Inductively coupled plasma mass spectrometry

Based on a previous methodology for database collection [6], an Excel-based data collection template was generated to standardise and collect the denomination and characteristics of digestates, as well as the metadata associated with the AD operational conditions, feedstocks composition and post-treatments leading to 7 Excel sheets for the French digestates collection and 5 Excel sheets for the Literature collection (Table S1 and S2, Supplementary Material).

Regarding the potential organic C and N mineralization from digestates and the fractionation dataset, organic carbon and nitrogen after 91 days of soil incubation under laboratory conditions (28°C, gravimetric water content equivalent to field capacity, pF 2.5) were measured following the standard method [7], in 3 replicates.

The Van Soest fractions of OM were obtained according to [8] and are used to calculate the IROC value, an indicator of the potential residual organic carbon in soils over the long term after application as defined in [4]. The ISBAMO parameters (OM fractionation and 3D fluorescence spectroscopy indicators) were obtained according to the analytical protocol described in [5], in 2 replicates.

The organic contaminants were measured in 3 replicates according to the methodology described in [9] concerning PAH ((BaA, benzo(a)anthracene; BaP, benzo(a)pyrene; BbF, benzo(b)fluoranthene; BkF, benzo(k) fluoranthene; Chry, chrysene; Flt, fluoranthene; Flu, fluorene; IPY, indeno(1,2,3,cd) pyrene; PHE, phenanthrene; PYR, pyrene, ANT, anthracene; NAP, naphthalene; DBA dibenzo(ah)anthracene; BPE: Benzo(ghi)perylene) and NP, and [10] for pharmaceutical compounds (ACTC, 5a,6-anhydrochlorotetracycline hydrochloride – ATC, 5a,6-anhydrotetracycline hydrochloride – DOX, doxycycline hyclate – IBU, ibuprofen – TC, tetracycline antibiotics – OFL, ofloxacin).

Statistics were carried out in R language version 4.2.1 [11]. Data wrangling, transformation and visualisation was performed using the following R packages: ggplot2 [12] version 3.4.0, forcats version 0.5.1 [13] and dplyr version 1.0.9 [14]. Tukey pairwise comparisons have been done using the multcomp package version 1.4-19 [15]. Statistical analysis was conducted with the following steps:

• Statistical factors: the main factors analyzed were the digestate fractions (i.e. raw, liquid, solid and compost), which were considered as independent variables. Additionally, the AD operational conditions and the feedstock categories were included as factors in the analyses, when applicable (Fig. 1, Fig. 2 respectively).
• Variables of response: the dependent variables measured included the physicochemical parameters (DM in % of raw matter, OM in g.kg^-1^ DM, N_tot in g.kg^-1^ DM, N_NH_4_ in g.kg^-1^ DM, K_2_O_total in g.kg^-1^ DM, P_2_O_5__total in g.kg^-1^ DM) as well as the potential C and N mineralization after 91 days of soil incubation (C91 in % of C and N91 in % of N_tot), IROC in % of OM, NH_4_/N_tot ratio and trace metal elements measured on digestates described in Supplementary Material (Table S1). Boxplots were created to describe the variability of each variable according to the factors.
• Significance tests: after verifying normality (Shapiro-Wilk test) and homoscedasticity (Bartlett test), the parametric ANOVA test was applied, followed by pairwise comparisons using Tukey's test. If the assumptions of normality and homoscedasticity were not met, the non-parametric Kruskal-Wallis test was applied. Letters were assigned and displayed on the plots only when significant differences were found.

## Limitations

The numerous data sources meant that variables were not always measured using the same analytical methods, and for some data, the analytical methods were missing. Reported standard analytical methods and data measured with non-standard or missing methods were compared to the standard using Tukey pairwise comparisons with the multcomp package (version 1.4-19) [15]. Across all variables, data measured with non-standard or missing methods were found to be statistically similar (p < 0.05) to those measured with standard methods. Therefore, all analytical methods were retained, except for the Dumas method for Total Nitrogen. The calcination required by this method increased the likelihood of volatilizing N_NH4, leading to an underestimation of total nitrogen. For datasets reporting organic contaminants and some other parameters, the number of observations was low, which limited the statistical analysis (e.g., Tukey pairwise comparison could not be applied).

## Ethics Statement

The authors have read and follow the ethical requirements for publication in Data in Brief and confirm that the current work does not involve human subjects, animal experiments, or any data collected from social media platforms*.*

## CRediT Author Statement

**Lucille Caradec:** Conceptualization, Methodology, Formal Analysis, Visualization, Writing-Original draft preparation. **Aurélia Michaud**: Conceptualization, Methodology, Resources, Validation, Data curation, Writing- Reviewing and Editing, Supervision. **Mariana Moreira**: Resources, Project administration, Writing- Reviewing and Editing. **Ivan Desneulin :** Resources, Data curation. **Sylvaine Berger:** Resources, Data curation, Writing- Reviewing and Editing. **Dominique Patureau**: Resources, Writing- Reviewing and Editing. **Sabine Houot:** Conceptualization, Validation, Writing- Reviewing and Editing, Supervision. **Florent Levavasseur:** Resources, Data curation, Writing- Reviewing and Editing. **Antoine Savoie**: Resources, Writing- Reviewing and Editing. **Julie Jimenez:** Conceptualization, Resources, Validation, Data curation, Writing-Original draft preparation, Supervision, Project administration.

## Data Availability

Data.gouv.frAgricultural digestates - database of physico-chemical properties and process informations (Original data).

## Appendix Supplementary materials

Image, application 1
