# Supplementary material for: Comprehensive dataset on the physicochemical characteristics of agrowastes digestates from anaerobic digestion
Source: Data Brief. 2025 Apr 8;60:111550. doi: 10.1016/j.dib.2025.111550 (PMC12175230; doi:10.1016/j.dib.2025.111550)
Supplement: Supplementary file 1 [file mmc1.docx]

**Supplementary data**

**Comprehensive Dataset on the Physicochemical Characteristics of Agrowastes Digestates from Anaerobic Digestion**

Lucille Caradec^a^, Aurélia Michaud^a^, Mariana Moreira^b^, Ivan Desneulin^c^, Sylvaine Berger^c^, Dominique Patureau^d^, Sabine Houot^e^, Florent Levavasseur^e^, Antoine Savoie^f^, Julie Jimenez*^d^

^a^ INRAE, Institut Agro-UMR SAS, 65 rue de St-Brieuc, CS 84215, 35042 Rennes, France

^b^ Chambre d’Agriculture de Bretagne – Rue Maurice Le Lannou 35000 RENNES, France

^c^ SOLAGRO – 75 voie du TOEC 31000 TOULOUSE, France

^d^ INRAE, Univ. Montpellier, LBE, 102 Avenue des étangs, 11100, Narbonne, France

^e^ INRAE, AgroParisTech, Université Paris-Saclay, UMR ECOSYS, 91120, Palaiseau, France

^f^ INRAE, UE PAO, 37380 Nouzilly, France

***Corresponding author :** [*julie.jimenez@inrae.fr*](mailto:julie.jimenez@inrae.fr)

**Table of content**

Table S1: Content of the related dataset (DOI: 10.57745/M1JSU5) concerning the French agricultural digestates collection 2

Table S2: Content of the related dataset (DOI: 10.57745/M1JSU5) concerning the Literature digestates collection 3

Table S3 : Statistics overview of the collected values for the main parameters physicochemical parameters for the French collection of digestates4

Table S4 : Statistics overview of the collected values for the metal trace elements for the French collection of digestates5

Table S5 : Statistics overview of the collected values for the organic contaminants for the French collection of digestates 6

Table S6 : Statistics overview of the collected values for the potential C and N mineralization as agonomical indicators and organic matter fractionation for French digestates collection7

Table S7 : Statistics overview of the collected values for the main parameters physicochemical parameters for the Literature collection8

Table S8 : Statistics overview of the collected values for the metal trace elements for the Literature collection9

Table S9 : References related to the digestates from the Literature collection10

Figure S1 : Boxplots obtained from “Trace Metals Elements composition” of the collected digestates dataset and comparison with European Regulation UE 2019/100913

Table S1: Content of the related dataset (DOI: 10.57745/M1JSU5) concerning the French agricultural digestates collection

| **Dataset name** | **Excel file name** | **Parameter Content** | **Digestates**  **number** |
| --- | --- | --- | --- |
| Physico-chemical characterization | French_Digestates_dataset_CNPK_20241220 | **Physico-chemical composition** in g/g dry matter:  dry matter (DM), organic matter concentration (OM), total carbon concentration (C_tot), total nitrogen concentration (N_tot), ammonia concentration (N_NH4), total potassium (K_2_O), total phosphorous concentration (P_2_O_5_), carbon to total nitrogen ratio (C/N), carbon to organic nitrogen ratio (C/Norg), ammonia on total nitrogen ratio (NH4/Ntot), total sodium concentration (Na_2_O), total calcium concentration (CaO), total magnesium concentration (MgO), total sulphur concentration (SO_3_) | **608** |
| Metals trace elements | French_Digestates_metallic_trace_elements_20241220 | **Metals trace elements concentrations** in mg/g of dry matter : silver (Ag), aluminium (Al), arsenic (As), bore (B), cadmium (Cd), cobalt (Co), chromium (Cr), copper (Co), iron (Fe), mercury (Hg), manganese (Mn), molybdate (Mo), nickel (Ni), lead (Pb), selenium (Se), thallium (Tl), zinc (Zn) | **155** |
| Organic trace contaminants | French_Digestates_organic_trace_contaminants_20241220 | **Organic trace contaminants concentrations** mg/g of dry matter: benzo(a)anthracene (BaA), benzo(a)pyrene (BaP), benzo(b)fluoranthene (BbF), benzo(k)fluoranthene (BkF), chrysene (Chry), fluoranthene (Flt), fluorine (Flu), indeno(1,2,3,cd) pyrene (IPY), phenanthrene (PHE), pyrene (PYR), anthracene (ANT), naphthalene (NAP), dibenzo(ah)anthracene (DBA), benzo(ghi)perylene (BPE), nonylphenol (NP), 5a,6-anhydrochlorotetracycline hydrochloride (ACTC), 5a,6-anhydrotetracycline hydrochloride (ATC), doxycycline hyclate (DOX), ibuprofen (IBU), tetracycline (TC), ofloxacin (OFL) | **45** |
| Potential C and N mineralization organic matter and fractionation | French_Digestates_OMfractionation_20241220 | **OM fractionation and soil organic C and N mineralization** according to Van Soest and ISBAMO (Jimenez et al., 2015), mineralized organic C and N after 91 days in soil (C91 and N91), Indicator of potential Residual Organic Carbon in percentage of organic matter (IROC) (Lashermes et al., 2009) | **535** |
| Process metadata | French_Digestates_process_20241220 | **Process conditions description** of AD (hydraulic retention time, temperature, feeding mode, phase separation, storage, post-treatment) and **feedstocks recipe** in raw mass percentage | **608** |
| Process glossary | French_Digestates_process_z_metadata_FR_EN_20241220 | **Glossary** of all the process conditions, post-treatments, feedstocks categories, and nomenclature in French and English | **-** |
| Parameters glossary | French_Digestates_dataset_z_metadata_FR_EN_20241220 | **Glossary of all the parameters** analyzed in the 4 analytical datasets and the related analytical methods described in French and English | **-** |

Table S2: Content of the related dataset (DOI: 10.57745/M1JSU5) concerning the Literature digestates collection

| **Dataset name** | **Excel file name** | **Parameter Content** | **Digestates**  **number** |
| --- | --- | --- | --- |
| Litterature Physico-chemical characterization | Publication_Digestates_dataset_CNPK_20241220 | **Physico-chemical composition** in g/g dry matter: dry matter (DM), organic matter concentration (OM), total carbon concentration (C_tot), total nitrogen concentration (N_tot), ammonia concentration (N_NH4), total potassium (K_2_O), total phosphorous concentration (P_2_O_5_), carbon to total nitrogen ratio (C/N), carbon to organic nitrogen ratio (C/Norg), ammonia on total nitrogen ratio (NH4/Ntot), total sodium concentration (Na_2_O), total calcium concentration (CaO), total magnesium concentration (MgO), total sulphur concentration (SO_3_) | **191** |
| Litterature supplementary_parameters | Publication_Digestates_dataset_supplementary_variables_20241220 | **Supplementary parameters:** trace elements (B, Cu, Cr, Fe, Mn, Zn) and fibers content (cellulose, hemicellulose and lignin) | **42** |
| Litterature process_metadata | Publication_Digestates_dataset_process_20241220 | **Process conditions description** of AD (hydraulic retention time, temperature, feeding mode, phase separation, storage, post-treatment) and **feedstocks recipe** in raw mass percentage | **198** |
| Literature process glossary | Publication_Digestates_process_z_metadata_FR_EN_20241220 | **Glossary** of all the process conditions, post-treatments, feedstocks categories, and nomenclature in French and English | **-** |
| Literature parameters glossary | Publication_Digestates_dataset_z_metadata_FR_EN_20241220 | **Glossary of all the parameters** analyzed in the 2 analytical datasets and the related analytical methods described in French and English | **-** |

Table S3 : Statistics overview of the collected values for the main parameters physicochemical parameters for the French collection of digestates

|  |  | **Raw (n=349)** | | | | | | | | | | **Liquid (n=126)** | | | | | | | | | | **Solid (n= 110)** | | | | | | | | | | **Compost (n=23)** | | | | | | | | | |  |
| --- | --- | --- | --- | --- | --- | --- | --- | --- | --- | --- | --- | --- | --- | --- | --- | --- | --- | --- | --- | --- | --- | --- | --- | --- | --- | --- | --- | --- | --- | --- | --- | --- | --- | --- | --- | --- | --- | --- | --- | --- | --- | --- |
|  |  | **Median** | | **1st**  **quartile** | | **3rd**  **quartile** | | **Mean** | | **CV** | | **Median** | | **1st**  **quartile** | | **3rd**  **quartile** | | **Mean** | | **CV** | | **Median** | | **1st**  **quartile** | | **3rd**  **quartile** | | **Mean** | | **CV** | | **Median** | | **1st**  **quartile** | | **3rd**  **quartile** | | **Mean** | | **CV** | |  |
| **DM** | % RM | | 7.80 | | 5.69 | | 10.20 | | 5.29 | | 60% | | 5.80 | | 4.30 | | 7.60 | | 2.80 | | 45% | | 24.71 | | 22.18 | | 28.69 | | 13.92 | | 50% | | 47.55 | | 40.53 | | 53.97 | | 18.00 | | 37% | |
| **OM** | g .kg TS^-1^ | | 677.00 | | 626.51 | | 715.63 | | 84.33 | | 13% | | 624.00 | | 571.22 | | 668.84 | | 81.47 | | 13% | | 824.00 | | 748.33 | | 854.14 | | 117.46 | | 15% | | 628.93 | | 493.26 | | 699.80 | | 127.74 | | 21% | |
| **C_total** | g C.kg TS^-1^ | | 353.10 | | 318.88 | | 381.84 | | 55.44 | | 16% | | 335.43 | | 299.89 | | 364.29 | | 62.94 | | 19% | | 416.28 | | 374.23 | | 435.98 | | 56.41 | | 14% | | 320.03 | | 278.56 | | 367.25 | | 62.35 | | 20% | |
| **N_tot** | g N.kg TS^-1^ | | 65.45 | | 48.74 | | 84.36 | | 49.38 | | 65% | | 84.98 | | 63.51 | | 102.95 | | 63.09 | | 66% | | 23.93 | | 19.39 | | 30.31 | | 9.44 | | 37% | | 27.86 | | 21.28 | | 32.06 | | 8.09 | | 29% | |
| **NH_4_** | g N.kg TS^-1^ | | 31.35 | | 16.85 | | 51.36 | | 37.07 | | 91% | | 44.82 | | 28.45 | | 62.08 | | 56.08 | | 101% | | 5.63 | | 2.32 | | 9.40 | | 5.37 | | 83% | | 4.09 | | 1.25 | | 6.18 | | 2.84 | | 72% | |
| **Norg** | g N.kg TS^-1^ | | 31.43 | | 25.04 | | 39.24 | | 23.56 | | 66% | | 39.09 | | 31.00 | | 46.80 | | 15.37 | | 38% | | 18.06 | | 15.05 | | 23.92 | | 7.44 | | 38% | | 20.90 | | 16.15 | | 25.13 | | 7.28 | | 33% | |
| **K_2_O** | g .kg TS^-1^ | | 57.53 | | 44.02 | | 70.49 | | 38.84 | | 60% | | 74.81 | | 56.95 | | 95.93 | | 52.58 | | 62% | | 18.00 | | 13.49 | | 23.26 | | 7.19 | | 40% | | 22.81 | | 21.27 | | 27.64 | | 5.91 | | 25% | |
| **P_2_O_5_** | g .kg TS^-1^ | | 27.80 | | 19.60 | | 41.27 | | 16.99 | | 53% | | 32.60 | | 24.98 | | 42.17 | | 24.01 | | 61% | | 22.73 | | 15.55 | | 30.38 | | 15.64 | | 60% | | 40.15 | | 31.39 | | 59.66 | | 34.90 | | 68% | |
| **pH** | - | | 8.02 | | 7.85 | | 8.40 | | 1.42 | | 18% | | 8.00 | | 7.82 | | 8.20 | | 0.28 | | 4% | | 8.98 | | 8.55 | | 9.14 | | 0.70 | | 8% | | 8.64 | | 7.16 | | 9.09 | | 1.02 | | 12% | |
| **C/N** | - | | 5.45 | | 3.90 | | 7.17 | | 3.63 | | 58% | | 4.14 | | 3.15 | | 5.40 | | 2.14 | | 48% | | 17.53 | | 14.19 | | 21.29 | | 5.85 | | 33% | | 11.50 | | 10.41 | | 12.45 | | 3.56 | | 29% | |
| **C/Norg** | - | | 11.17 | | 8.96 | | 14.54 | | 9.12 | | 70% | | 8.97 | | 6.91 | | 10.57 | | 17.02 | | 150% | | 22.86 | | 17.23 | | 27.36 | | 23.57 | | 90% | | 15.13 | | 11.37 | | 17.42 | | 5.57 | | 36% | |
| **NH_4_/Ntot** | - | | 0.51 | | 0.35 | | 0.62 | | 0.20 | | 40% | | 0.55 | | 0.44 | | 0.64 | | 0.16 | | 31% | | 0.25 | | 0.11 | | 0.36 | | 0.17 | | 69% | | 0.17 | | 0.09 | | 0.22 | | 0.11 | | 72% | |
| **Na_2_O** | g .kg TS^-1^ | | 8.00 | | 4.60 | | 21.21 | | 20.97 | | 131% | | 10.59 | | 7.90 | | 17.70 | | 8.42 | | 65% | | 2.80 | | 2.32 | | 4.40 | | 2.27 | | 62% | | 4.67 | | 4.67 | | 4.67 | | na | | na | |
| **CaO** | g .kg TS^-1^ | | 35.00 | | 29.22 | | 43.25 | | 20.35 | | 51% | | 44.77 | | 35.58 | | 52.64 | | 25.78 | | 52% | | 24.10 | | 18.37 | | 32.20 | | 12.73 | | 46% | | 56.98 | | 46.69 | | 67.00 | | 17.13 | | 30% | |
| **MgO** | g .kg TS^-1^ | | 9.70 | | 6.78 | | 13.52 | | 9.18 | | 81% | | 10.91 | | 7.21 | | 12.86 | | 5.65 | | 53% | | 8.38 | | 5.93 | | 11.01 | | 5.90 | | 62% | | 13.98 | | 13.23 | | 14.73 | | 10.40 | | 61% | |
| **SO_3_** | g .kg TS^-1^ | | 11.24 | | 5.42 | | 16.20 | | 7.07 | | 59% | | 16.00 | | 15.10 | | 25.20 | | 8.10 | | 41% | | 11.66 | | 10.00 | | 13.40 | | 4.31 | | 38% | | 16.44 | | 16.03 | | 17.03 | | 1.79 | | 11% | |

Table S4 : Statistics overview of the collected values for the metal trace elements for the French collection of digestates

|  |  | **Raw (n=93)** | | | | | **Liquid (n=31)** | | | | | **Solid (n=27)** | | | | | **Compost (n=4)** | | | | |
| --- | --- | --- | --- | --- | --- | --- | --- | --- | --- | --- | --- | --- | --- | --- | --- | --- | --- | --- | --- | --- | --- |
| mg .kg DM^-1^ | **RUE*** | **Median** | **1st**  **quartile** | **3rd**  **quartile** | **Mean** | **CV** | **Median** | **1st**  **quartile** | **3rd**  **quartile** | **Mean** | **CV** | **Median** | **1st**  **quartile** | **3rd**  **quartile** | **Mean** | **CV** | **Median** | **1st**  **quartile** | **3rd**  **quartile** | **Mean** | **CV** |
| **Ag** |  | 0.10 | 0.09 | 0.14 | 0.28 | 152% | 0.33 | 0.23 | 0.76 | 0.55 | 103% | 0.13 | 0.10 | 0.25 | 0.20 | 83% | 0.02 | 0.02 | 0.02 | 0.02 | na |
| **Al** |  | 3521.67 | 2125.00 | 5186.00 | 3833.73 | 62% | 6059.50 | 4373.25 | 7745.75 | 6059.50 | 79% | 3298.00 | 2423.50 | 5786.00 | 4373.67 | 80% | 13073.00 | 13073.00 | 13073.00 | 13073.00 | na |
| **As** | 40 | 1.90 | 1.25 | 2.60 | 3.27 | 145% | 1.75 | 1.43 | 2.12 | 2.06 | 79% | 1.12 | 0.87 | 1.32 | 1.14 | 54% | 2.42 | 2.16 | 2.67 | 2.42 | 30% |
| **B** |  | 32.70 | 24.50 | 51.09 | 37.69 | 43% | 36.03 | 34.22 | 38.94 | 36.25 | 21% | 21.52 | 17.13 | 31.00 | 23.71 | 39% | na | na | na | na | na |
| **Cd** | 1.5 | 0.46 | 0.30 | 0.56 | 0.48 | 61% | 0.60 | 0.36 | 0.75 | 1.25 | 170% | 0.24 | 0.17 | 0.39 | 0.36 | 98% | 0.40 | 0.36 | 0.45 | 0.40 | 34% |
| **Co** |  | 2.80 | 1.66 | 5.00 | 7.73 | 353% | 2.58 | 2.36 | 2.66 | 2.52 | 8% | 1.56 | 1.48 | 1.73 | 1.56 | 16% | 2.73 | 2.73 | 2.73 | 2.73 | na |
| **Cr** |  | 8.02 | 2.61 | 16.26 | 10.85 | 106% | 6.54 | 0.72 | 14.60 | 12.06 | 166% | 7.00 | 2.09 | 16.97 | 9.96 | 89% | 11.40 | 8.50 | 16.88 | 13.98 | 61% |
| **Cu** | 300 | 82.40 | 45.90 | 157.57 | 105.91 | 68% | 83.48 | 65.53 | 135.83 | 118.29 | 78% | 43.70 | 31.30 | 68.10 | 67.25 | 88% | 161.34 | 98.18 | 221.46 | 158.30 | 50% |
| **Fe** |  | 3750.00 | 2666.00 | 9965.96 | 8321.65 | 111% | 10650.00 | 4700.00 | 28478.23 | 19760.08 | 93% | 8480.86 | 5753.79 | 12858.04 | 11208.11 | 81% | 8956.62 | 8956.62 | 8956.62 | 8956.62 | na |
| **Hg** | 1 | 0.11 | 0.04 | 0.30 | 1.34 | 240% | 0.12 | 0.09 | 0.66 | 0.46 | 140% | 0.34 | 0.24 | 0.60 | 0.50 | 96% | na | na | na | na | na |
| **Mn** |  | 329.10 | 241.44 | 443.15 | 352.35 | 51% | 363.97 | 316.04 | 523.40 | 380.61 | 39% | 177.65 | 142.29 | 254.16 | 240.85 | 64% | 909.24 | 909.24 | 909.24 | 909.24 | na |
| **Mo** |  | 3.53 | 2.75 | 5.47 | 5.43 | 149% | 4.07 | 2.75 | 5.96 | 4.83 | 58% | 1.81 | 1.33 | 2.97 | 2.27 | 62% | 4.40 | 3.88 | 4.75 | 4.22 | 23% |
| **Ni** | 50 | 9.90 | 7.80 | 14.35 | 11.94 | 62% | 10.70 | 6.92 | 14.83 | 12.21 | 45% | 7.96 | 4.47 | 11.79 | 9.50 | 66% | 11.77 | 7.38 | 15.80 | 11.41 | 50% |
| **Pb** | 120 | 4.69 | 3.48 | 8.35 | 7.92 | 112% | 5.55 | 4.81 | 10.60 | 12.07 | 143% | 4.69 | 2.54 | 8.70 | 9.79 | 166% | 2.55 | 1.60 | 4.65 | 3.70 | 88% |
| **Se** |  | 3.20 | 1.70 | 10.43 | 10.87 | 157% | 2.00 | 1.60 | 2.78 | 3.18 | 111% | 2.00 | 1.50 | 4.80 | 3.36 | 76% | 2.85 | 2.48 | 3.23 | 2.85 | 37% |
| **Ti** |  | 47.09 | 0.03 | 212.94 | 183.93 | 148% | 325.57 | 163.72 | 375.84 | 251.19 | 88% | 130.43 | 97.33 | 345.04 | 251.44 | 107% | 852.04 | 852.04 | 852.04 | 852.04 | na |
| **Zn** | 800 | 278.00 | 202.04 | 562.89 | 450.83 | 81% | 366.77 | 240.50 | 502.94 | 486.70 | 103% | 164.55 | 115.00 | 200.00 | 311.33 | 125% | 578.89 | 429.48 | 766.35 | 616.94 | 46% |

*Thresholds for organic fertilizer regulation- UE 2019/1009 - PFC1-A

Table S5 : Statistics overview of the collected values for the organic contaminants for the French collection of digestates

*(Thresholds for compost and digestate regulation- UE 2019/1009 : sum of HAP_16_ <6 000 µg/kgDM^-1^)*

|  | **Raw (n=26)** | | | | | **Liquid (n=8)** | | | | | **Solid (n=9)** | | | | | **Compost (n=1)** | | | |
| --- | --- | --- | --- | --- | --- | --- | --- | --- | --- | --- | --- | --- | --- | --- | --- | --- | --- | --- | --- |
| µg .kg DM^-1^ | **Median** | **1st**  **quartile** | **3rd**  **quartile** | **Mean** | **CV** | **Median** | **1st**  **quartile** | **3rd**  **quartile** | **Mean** | **CV** | **Median** | **1st**  **quartile** | **3rd**  **quartile** | **Mean** | **CV** | **Median** | **1st**  **quartile** | **3rd**  **quartile** | **Mean** |
| **BaA** | 8.00 | 1.00 | 23.00 | 37.22 | 220% | 52.00 | 48.50 | 141.75 | 109.50 | 97% | 14.00 | 7.00 | 32.00 | 76.10 | 182% | 4.00 | 4.00 | 4.00 | 4.00 |
| **BaP** | 44.50 | 7.00 | 193.25 | 106.93 | 133% | 52.00 | 34.75 | 91.75 | 74.50 | 99% | 36.00 | 9.00 | 50.00 | 73.90 | 148% | 6.00 | 6.00 | 6.00 | 6.00 |
| **BbF** | 60.00 | 12.50 | 215.00 | 150.77 | 115% | 42.00 | 16.00 | 61.00 | 83.90 | 140% | 45.00 | 24.75 | 146.38 | 126.13 | 147% | na | na | na | na |
| **BkF** | 9.00 | 2.00 | 13.00 | 17.61 | 181% | 26.00 | 18.00 | 107.25 | 74.83 | 132% | 13.00 | 7.50 | 130.00 | 87.33 | 158% | 2.00 | 2.00 | 2.00 | 2.00 |
| **CHR** | 7.50 | 3.00 | 19.50 | 34.55 | 209% | 52.00 | 32.00 | 145.00 | 125.00 | 133% | 18.50 | 6.00 | 37.00 | 102.00 | 187% | 6.00 | 6.00 | 6.00 | 6.00 |
| **FLT** | 29.00 | 16.00 | 90.50 | 105.09 | 188% | 89.00 | 41.50 | 133.00 | 211.40 | 148% | 49.50 | 27.00 | 57.00 | 191.70 | 178% | 1.00 | 1.00 | 1.00 | 1.00 |
| **FLU** | 85.00 | 16.63 | 151.20 | 121.01 | 122% | 45.00 | 27.75 | 94.50 | 61.67 | 77% | 40.50 | 16.00 | 67.50 | 48.71 | 90% | 3.00 | 3.00 | 3.00 | 3.00 |
| **IPY** | 73.50 | 21.25 | 160.83 | 210.67 | 184% | 31.00 | 17.00 | 120.00 | 81.00 | 138% | 156.75 | 84.88 | 228.63 | 156.75 | 130% | na | na | na | na |
| **PHE** | 55.00 | 28.50 | 139.00 | 109.79 | 136% | 95.25 | 68.88 | 180.50 | 197.75 | 123% | 42.00 | 34.50 | 92.25 | 137.86 | 163% | 26.00 | 26.00 | 26.00 | 26.00 |
| **PYR** | 43.25 | 31.38 | 114.75 | 122.02 | 153% | 48.00 | 22.50 | 116.00 | 146.20 | 154% | 62.50 | 41.00 | 136.50 | 155.58 | 139% | na | na | na | na |
| **ANT** | 6.00 | 1.75 | 10.38 | 19.00 | 207% | 67.50 | 42.25 | 92.75 | 67.50 | 106% | 9.00 | 2.00 | 21.00 | 35.90 | 172% | na | na | na | na |
| **NAP** | 23.63 | 12.38 | 33.44 | 22.19 | 81% | na | na | na | na | na | 67.50 | 67.50 | 67.50 | 67.50 | na | na | na | na | na |
| **DBA** | 1.00 | 0.25 | 52.63 | 58.95 | 233% | 34.75 | 20.88 | 48.63 | 34.75 | 113% | 3.00 | 2.00 | 47.50 | 32.00 | 162% | 0.00 | 0.00 | 0.00 | 0.00 |
| **BPE** | 10.00 | 4.00 | 11.00 | 25.22 | 162% | 76.00 | 47.00 | 125.50 | 89.67 | 89% | 32.00 | 12.50 | 96.75 | 77.25 | 137% | 8.00 | 8.00 | 8.00 | 8.00 |
| **NP** | 619.88 | 490.92 | 2593.75 | 2464.79 | 156% | na | na | na | na | na | na | na | na | na | na | na | na | na | na |
| **NP1EO** | 1259.29 | 595.50 | 3718.00 | 7010.00 | 203% | 1658.00 | 1658.00 | 1658.00 | 1658.00 | na | 21896.00 | 13059.50 | 30732.50 | 21896.00 | 114% | na | na | na | na |
| **NP2EO** | 2556.28 | 1837.22 | 7843.67 | 7124.60 | 136% | na | na | na | na | na | na | na | na | na | na | 866.00 | 866.00 | 866.00 | 866.00 |
| **ACTC** | 900.00 | 600.62 | 1148.87 | 901.97 | 40% | 5616.16 | 3183.08 | 8049.23 | 5616.16 | 123% | 1752.03 | 1126.02 | 2764.05 | 2009.37 | 82% | 3153.90 | 3153.90 | 3153.90 | 3153.90 |
| **ATC** | 300.00 | 300.00 | 927.27 | 590.14 | 81% | 1724.56 | 1237.28 | 2211.85 | 1724.56 | 80% | 300.00 | 250.00 | 621.62 | 481.08 | 84% | 1785.61 | 1785.61 | 1785.61 | 1785.61 |
| **DOX** | 1864.77 | 1107.39 | 6832.26 | 4671.51 | 133% | 3625.37 | 2037.69 | 5213.06 | 3625.37 | 124% | 2277.27 | 1853.99 | 5506.13 | 4147.66 | 96% | na | na | na | na |
| **IBU** | 78.13 | 43.44 | 335.92 | 301.23 | 160% | 67.57 | 61.16 | 73.98 | 67.57 | 27% | 33.85 | 29.42 | 38.85 | 34.24 | 28% | 52.35 | 52.35 | 52.35 | 52.35 |
| **TC** | 366.17 | 245.58 | 1026.69 | 726.13 | 116% | 803.62 | 597.98 | 1009.26 | 803.62 | 72% | 125.00 | 120.00 | 169.99 | 151.66 | 36% | 43.65 | 43.65 | 43.65 | 43.65 |
| **OFL** | 16.25 | 13.13 | 530.64 | 527.51 | 195% | 1334.01 | 1334.01 | 1334.01 | 1334.01 | na | 663.78 | 340.64 | 986.91 | 663.78 | 138% | na | na | na | na |

Table S6 : Statistics overview of the collected values for the potential C and N mineralization as agronomical indicators and organic matter fractionation for French digestates collection

|  |  | **Raw (311)** | | | | | **Liquid (113)** | | | | | **Solid (90)** | | | | | **Compost (21)** | | | | |
| --- | --- | --- | --- | --- | --- | --- | --- | --- | --- | --- | --- | --- | --- | --- | --- | --- | --- | --- | --- | --- | --- |
|  |  | **Median** | **1st**  **quartile** | **3rd**  **quartile** | **Mean** | **CV** | **Median** | **1st quartile** | **3rd quartile** | **Mean** | **CV** | **Median** | **1st quartile** | **3rd**  **quartile** | **Mean** | **CV** | **Median** | **1st quartile** | **3rd quartile** | **Mean** | **CV** |
| **C91** | %C | 32,00 | 25,25 | 42,29 | 34,60 | 40% | 31,64 | 24,63 | 36,67 | 34,34 | 44% | 27,00 | 21,65 | 36,93 | 29,68 | 45% | 13,58 | 12,29 | 14,86 | 13,58 | 27% |
| **IROC** | %OM | 58,57 | 47,15 | 65,35 | 55,58 | 26% | 62,70 | 46,17 | 69,53 | 58,13 | 31% | 61,58 | 57,32 | 66,95 | 61,22 | 15% | 79,40 | 79,40 | 79,40 | 79,40 | na |
| **N91** | %Norg | 16,00 | 6,08 | 30,75 | 18,56 | 138% | 40,50 | 24,52 | 58,25 | 41,66 | 50% | 1,79 | -8,76 | 15,50 | 5,20 | 410% | 4,50 | 0,25 | 8,75 | 4,50 | 267% |
| **COD** | mg .kg DM^-1^ | 1146,87 | 1005,90 | 1293,34 | 1172,78 | 21% | 995,42 | 893,75 | 1086,42 | 1040,97 | 21% | 1234,26 | 1132,40 | 1288,28 | 1184,44 | 12% | 812,26 | 787,81 | 888,17 | 840,75 | 20% |
| **SPOM** | %COD | 11,77 | 3,75 | 20,35 | 14,01 | 87% | 28,98 | 23,57 | 40,37 | 31,54 | 55% | na | na | na | na | na | na | na | na | na | na |
| **REOM** | %COD | 4,13 | 2,90 | 6,64 | 5,37 | 79% | 3,28 | 2,08 | 4,19 | 3,30 | 48% | 3,56 | 2,70 | 4,09 | 3,48 | 34% | 2,65 | 1,85 | 4,75 | 3,90 | 76% |
| **DOM** | %COD | 3,90 | 2,77 | 6,17 | 5,38 | 84% | 3,04 | 2,00 | 3,72 | 3,00 | 49% | 2,68 | 2,03 | 3,71 | 2,90 | 64% | 4,27 | 3,35 | 5,93 | 4,31 | 45% |
| **SEOM** | %COD | 12,16 | 9,06 | 15,45 | 12,84 | 46% | 10,80 | 6,86 | 16,98 | 11,62 | 50% | 11,27 | 7,98 | 15,50 | 12,31 | 40% | 16,40 | 14,65 | 21,49 | 17,77 | 39% |
| **PEOM** | %COD | 26,20 | 19,13 | 35,46 | 28,00 | 42% | 17,72 | 9,80 | 26,10 | 19,50 | 62% | 40,29 | 34,23 | 44,58 | 40,45 | 28% | 35,79 | 19,52 | 44,30 | 37,20 | 48% |
| **NEOM** | %COD | 34,44 | 26,65 | 45,12 | 34,92 | 45% | 30,35 | 16,98 | 41,77 | 30,96 | 57% | 41,40 | 34,30 | 46,51 | 40,87 | 30% | 41,81 | 27,20 | 48,38 | 36,83 | 49% |
| **DOM_N** | %Ntot | 27,86 | 11,59 | 41,49 | 27,17 | 62% | 64,05 | 50,12 | 77,00 | 59,54 | 44% | na | na | na | na | na | na | na | na | na | na |
| **SPOM_N** | %Ntot | 8,62 | 0,45 | 15,20 | 8,92 | 76% | 5,25 | 2,84 | 7,48 | 5,60 | 57% | 18,93 | 15,26 | 22,72 | 18,66 | 34% | 14,68 | 8,60 | 22,58 | 16,82 | 67% |
| **REOM_N** | %Ntot | 3,84 | 2,06 | 4,93 | 4,45 | 97% | 3,20 | 1,67 | 5,23 | 3,46 | 66% | 8,94 | 6,24 | 12,06 | 9,24 | 34% | 7,88 | 7,19 | 8,58 | 7,74 | 24% |
| **SEOM_N** | %Ntot | 3,80 | 3,24 | 4,77 | 4,81 | 65% | 8,06 | 3,70 | 12,02 | 9,37 | 77% | 17,04 | 14,99 | 22,27 | 18,60 | 30% | 20,11 | 18,98 | 23,70 | 20,09 | 25% |
| **PEOM_N** | %Ntot | 36,77 | 21,95 | 53,44 | 35,68 | 57% | 10,97 | 3,69 | 17,28 | 11,55 | 73% | 16,34 | 11,38 | 19,96 | 15,04 | 47% | 17,61 | 15,86 | 20,56 | 16,32 | 50% |
| **NEOM_N** | %Ntot | 9,11 | 5,92 | 16,34 | 12,88 | 76% | 13,42 | 3,10 | 19,39 | 13,93 | 94% | 33,15 | 24,62 | 50,86 | 37,22 | 52% | 41,02 | 31,37 | 52,78 | 43,64 | 34% |

Table S7 : Statistics overview of the collected values for the main parameters physicochemical parameters for the Literature collection

|  |  | **Raw (121)** | | | | | | **Liquid (36)** | | | | | **Solid (35)** | | | | |  |  |
| --- | --- | --- | --- | --- | --- | --- | --- | --- | --- | --- | --- | --- | --- | --- | --- | --- | --- | --- | --- |
|  |  | **Median** | | **1st**  **quartile** | **3rd**  **quartile** | **Mean** | **CV** | **Median** | **1st**  **quartile** | **3rd**  **quartile** | **Mean** | **CV** | **Median** | **1st**  **quartile** | **3rd**  **quartile** | **Mean** | **CV** | | |
| **DM** | % RM | | 4.45 | 2.18 | 6.50 | 6.55 | 127% | 3.80 | 2.10 | 5.20 | 7.41 | 222% | 25.75 | 20.75 | 29.43 | 24.72 | 29% | |  |
| **OM** | g .kg TS^-1^ | | 676.70 | 619.25 | 735.23 | 657.36 | 16% | 633.90 | 564.75 | 658.70 | 608.87 | 22% | 843.00 | 820.95 | 882.00 | 816.20 | 14% | |  |
| **C_total** | g C.kg TS^-1^ | | 338.35 | 309.63 | 367.61 | 328.68 | 16% | 316.95 | 282.38 | 329.35 | 304.44 | 22% | 421.50 | 410.48 | 441.00 | 408.10 | 14% | |  |
| **N_tot** | g N.kg TS^-1^ | | 67.80 | 46.73 | 115.85 | 88.23 | 68% | 67.00 | 43.85 | 121.80 | 99.91 | 93% | 28.00 | 23.45 | 31.00 | 27.22 | 19% | |  |
| **NH_4_** | g N.kg TS^-1^ | | 32.41 | 17.62 | 80.62 | 54.65 | 96% | 32.90 | 12.23 | 45.48 | 48.61 | 140% | 6.70 | 6.20 | 7.80 | 7.02 | 31% | |  |
| **Norg** | g N.kg TS^-1^ | | 31.30 | 23.90 | 35.76 | 32.15 | 58% | 41.58 | 32.30 | 67.95 | 54.90 | 75% | 19.80 | 16.80 | 20.40 | 18.72 | 14% | |  |
| **K_2_O** | g .kg TS^-1^ | | 68.08 | 33.32 | 90.16 | 63.93 | 62% | na | na | na | na | na | na | na | na | na | na | |  |
| **P_2_O_5_** | g .kg TS^-1^ | | 23.93 | 20.61 | 53.59 | 38.49 | 76% | 41.68 | 39.96 | 43.74 | 41.91 | 9% | na | na | na | na | na | |  |
| **pH** | - | | 7.90 | 7.60 | 8.30 | 7.87 | 7% | 8.20 | 7.75 | 8.30 | 8.09 | 7% | 8.80 | 8.35 | 9.10 | 8.73 | 6% | |  |
| **C/N** | - | | 5.91 | 3.21 | 8.32 | 6.81 | 86% | 4.54 | 2.35 | 8.39 | 7.26 | 111% | 15.56 | 13.83 | 16.87 | 16.22 | 29% | |  |
| **C/Norg** | - | | 11.59 | 9.42 | 15.41 | 17.49 | 102% | 6.21 | 3.87 | 10.49 | 9.48 | 93% | 21.11 | 20.32 | 21.98 | 21.32 | 7% | |  |
| **NH_4_/Ntot** | - | | 0.53 | 0.37 | 0.70 | 0.52 | 41% | 0.39 | 0.27 | 0.50 | 0.39 | 42% | 0.27 | 0.24 | 0.28 | 0.27 | 25% | |  |
| **Na_2_O** | g .kg TS^-1^ | | 28.96 | 16.57 | 40.10 | 28.16 | 53% | na | na | na | na | na | na | na | na | na | na | |  |
| **CaO** | g .kg TS^-1^ | | 56.30 | 33.36 | 63.11 | 51.54 | 46% | na | na | na | na | na | na | na | na | na | na | |  |
| **MgO** | g .kg TS^-1^ | | 174.80 | 75.86 | 256.14 | 180.95 | 65% | na | na | na | na | na | na | na | na | na | na | |  |
| **SO_3_** | g .kg TS^-1^ | | 24.00 | 14.19 | 25.88 | 25.42 | 70% | na | na | na | na | na | na | na | na | na | na | |  |

Table S8 : Statistics overview of the collected values for the metal trace elements for the Literature collection

|  |  | **Raw (n=19)** | | | | | **Liquid (n=7)** | | | | | **Solid (n=16)** | | | | |
| --- | --- | --- | --- | --- | --- | --- | --- | --- | --- | --- | --- | --- | --- | --- | --- | --- |
| mg .kg DM^-1^ | **RUE*** | **Median** | **1st**  **quartile** | **3rd**  **quartile** | **Mean** | **CV** | **Median** | **1st**  **quartile** | **3rd**  **quartile** | **Mean** | **CV** | **Median** | **1st**  **quartile** | **3rd**  **quartile** | **Mean** | **CV** |
| **B** |  | 78,75 | 68,73 | 104,88 | 89,39 | 47% |  |  |  |  |  |  |  |  |  |  |
| **Cu** | 300 | 208,30 | 156,35 | 554,18 | 530,76 | 180% | 775,50 | 689,45 | 778,05 | 719,83 | 14% | 17,90 | 15,10 | 22,40 | 19,03 | 39% |
| **Cr** |  | 69,25 | 25,63 | 112,68 | 69,05 | 87% | 266,70 | 141,90 | 314,10 | 215,10 | 83% | 27,50 | 24,15 | 32,20 | 28,40 | 28% |
| **Fe** |  | 2350,00 | 2100,00 | 3450,00 | 2957,14 | 62% |  |  |  |  |  |  |  |  |  |  |
| **Mn** |  | 331,45 | 232,65 | 521,90 | 396,34 | 64% |  |  |  |  |  |  |  |  |  |  |
| **Zn** | 800 | 472,60 | 307,40 | 1578,90 | 1145,70 | 121% | 51,20 | 45,65 | 87,50 | 71,70 | 63% | 1,80 | 1,55 | 2,00 | 1,77 | 26% |

*Thresholds for organic fertilizer regulation- UE 2019/1009 - PFC1-A

Table S9 : References related to the digestates from the Literature collection

| **Literature collection : peer-reviewed scientific articles** |
| --- |
| Akhiar, A., Battimelli, A., Torrijos, M., Carrere, H., 2017. Comprehensive characterization of the liquid fraction of digestates from full-scale anaerobic co-digestion. Waste Manag. 59, 118–128. https://doi.org/10.1016/j.wasman.2016.11.005 |
| Alburquerque, J.A., de la Fuente, C., Campoy, M., Carrasco, L., Nájera, I., Baixauli, C., Caravaca, F., Roldán, A., Cegarra, J., Bernal, M.P., 2012. Agricultural use of digestate for horticultural crop production and improvement of soil properties. Eur. J. Agron. 43, 119–128. https://doi.org/10.1016/j.eja.2012.06.001 |
| Alburquerque, J.A., de la Fuente, C., Ferrer-Costa, A., Carrasco, L., Cegarra, J., Abad, M., Bernal, M.P., 2012. Assessment of the fertiliser potential of digestates from farm and agroindustrial residues. Biomass and Bioenergy 40, 181–189. https://doi.org/10.1016/J.BIOMBIOE.2012.02.018 |
| Ali, A.M., Nesse, A.S., Eich-Greatorex, S., Sogn, T.A., Aanrud, S.G., Aasen Bunæs, J.A., Lyche, J.L., Kallenborn, R., 2019. Organic contaminants of emerging concern in Norwegian digestates from biogas production. Environ. Sci. Process. Impacts 21, 1498–1508. https://doi.org/10.1039/C9EM00175A |
| Bachmann, S., Wentzel, S., Eichler‐Löbermann, B., 2011. Codigested dairy slurry as a phosphorus and nitrogen source for *Zea mays* L. and *Amaranthus cruentus* L. J. Plant Nutr. Soil Sci. 174, 908–915. https://doi.org/10.1002/jpln.201000383 |
| Bres, P., Beily, M.E., Young, B.J., Gasulla, J., Butti, M., Crespo, D., Candal, R., Komilis, D., 2018. Performance of semi-continuous anaerobic co-digestion of poultry manure with fruit and vegetable waste and analysis of digestate quality: A bench scale study. Waste Manag. 82, 276–284. https://doi.org/10.1016/j.wasman.2018.10.041 |
| Cavalli, D., Cabassi, G., Borrelli, L., Geromel, G., Bechini, L., Degano, L., Marino Gallina, P., 2016. Nitrogen fertilizer replacement value of undigested liquid cattle manure and digestates. Eur. J. Agron. 73, 34–41. https://doi.org/10.1016/j.eja.2015.10.007 |
| Cerda, A., Mejias, L., Rodríguez, P., Rodríguez, A., Artola, A., Font, X., Gea, T., Sánchez, A., 2019. Valorisation of digestate from biowaste through solid-state fermentation to obtain value added bioproducts: A first approach. Bioresour. Technol. 271, 409–416. https://doi.org/10.1016/j.biortech.2018.09.131 |
| Chantigny, M.H., Angers, D.A., Bélanger, G., Rochette, P., Eriksen‐Hamel, N., Bittman, S., Buckley, K., Massé, D., Gasser, M., 2008. Yield and Nutrient Export of Grain Corn Fertilized with Raw and Treated Liquid Swine Manure. Agron. J. 100, 1303–1309. https://doi.org/10.2134/agronj2007.0361 |
| Comino, E., Riggio, V.A., Rosso, M., 2012. Biogas production by anaerobic co-digestion of cattle slurry and cheese whey. Bioresour. Technol. 114, 46–53. https://doi.org/10.1016/j.biortech.2012.02.090 |
| De Moor, S., Velghe, F., Wierinck, I., Michels, E., Ryckaert, B., De Vocht, A., Verbeke, W., Meers, E., 2013. Feasibility of grass co-digestion in an agricultural digester, influence on process parameters and residue composition. Bioresour. Technol. 150, 187–194. https://doi.org/10.1016/j.biortech.2013.10.011 |
| Elalami, D., Monlau, F., Carrere, H., Abdelouahdi, K., Oukarroum, A., Zeroual, Y., Barakat, A., 2020. Effect of coupling alkaline pretreatment and sewage sludge co-digestion on methane production and fertilizer potential of digestate. Sci. Total Environ. 743, 140670. https://doi.org/10.1016/j.scitotenv.2020.140670 |
| Fouda, S., von Tucher, S., Lichti, F., Schmidhalter, U., 2013. Nitrogen availability of various biogas residues applied to ryegrass. J. Plant Nutr. Soil Sci. 176, 572–584. https://doi.org/10.1002/jpln.201100233 |
| Iocoli, G.A., Zabaloy, M.C., Pasdevicelli, G., Gómez, M.A., 2019. Use of biogas digestates obtained by anaerobic digestion and co-digestion as fertilizers: Characterization, soil biological activity and growth dynamic of Lactuca sativa L. Sci. Total Environ. 647, 11–19. https://doi.org/10.1016/j.scitotenv.2018.07.444 |
| Loria, E.R., Sawyer, J.E., 2005. Extractable Soil Phosphorus and Inorganic Nitrogen following Application of Raw and Anaerobically Digested Swine Manure. Agron. J. 97, 879–885. https://doi.org/10.2134/agronj2004.0249 |
| Loria, E.R., Sawyer, J.E., Barker, D.W., Lundvall, J.P., Lorimor, J.C., 2007. Use of Anaerobically Digested Swine Manure as a Nitrogen Source in Corn Production. Agron. J. 99, 1119–1129. https://doi.org/10.2134/agronj2006.0251 |
| Marcato, C.E., Pinelli, E., Pouech, P., Winterton, P., Guiresse, M., 2008. Particle size and metal distributions in anaerobically digested pig slurry. Bioresour. Technol. 99, 2340–2348. https://doi.org/10.1016/j.biortech.2007.05.013 |
| Massaccesi, L., Sordi, A., Micale, C., Cucina, M., Zadra, C., Di Maria, F., Gigliotti, G., 2013. Chemical characterisation of percolate and digestate during the hybrid solid anaerobic digestion batch process. Process Biochem. 48, 1361–1367. https://doi.org/10.1016/j.procbio.2013.06.026 |
| Massé, D.I., Croteau, F., Masse, L., 2007. The fate of crop nutrients during digestion of swine manure in psychrophilic anaerobic sequencing batch reactors. Bioresour. Technol. 98, 2819–2823. https://doi.org/10.1016/j.biortech.2006.07.040 |
| Menardo, S., Balsari, P., Dinuccio, E., Gioelli, F., 2011. Thermal pre-treatment of solid fraction from mechanically-separated raw and digested slurry to increase methane yield. Bioresour. Technol. 102, 2026–2032. https://doi.org/10.1016/j.biortech.2010.09.067 |
| Möller, K., Stinner, W., Deuker, A., Leithold, G., 2008. Effects of different manuring systems with and without biogas digestion on nitrogen cycle and crop yield in mixed organic dairy farming systems. Nutr. Cycl. Agroecosystems 82, 209–232. https://doi.org/10.1007/s10705-008-9196-9 |
| Peters, K., Jensen, L.S., 2011. Biochemical characteristics of solid fractions from animal slurry separation and their effects on C and N mineralisation in soil. Biol. Fertil. Soils 47, 447–455. https://doi.org/10.1007/s00374-011-0550-8 |
| PETERSEN, J., SØRENSEN, P., 2008. Loss of nitrogen and carbon during storage of the fibrous fraction of separated pig slurry and influence on nitrogen availability. J. Agric. Sci. 146, 403–413. https://doi.org/10.1017/S0021859607007654 |
| Pognani, M., D’Imporzano, G., Scaglia, B., Adani, F., 2009. Substituting energy crops with organic fraction of municipal solid waste for biogas production at farm level: A full-scale plant study. Process Biochem. 44, 817–821. https://doi.org/10.1016/j.procbio.2009.03.014 |
| Rico, C., Muñoz, N., Rico, J.L., 2015. Anaerobic co-digestion of cheese whey and the screened liquid fraction of dairy manure in a single continuously stirred tank reactor process: Limits in co-substrate ratios and organic loading rate. Bioresour. Technol. 189, 327–333. https://doi.org/10.1016/j.biortech.2015.04.032 |
| Rubæk, G.H., Henriksen, K., Petersen, J., Rasmussen, B., Sommer, S.G., 1996. Effects of application technique and anaerobic digestion on gaseous nitrogen loss from animal slurry applied to ryegrass ( *Lolium perenne* ). J. Agric. Sci. 126, 481–492. https://doi.org/10.1017/S0021859600075572 |
| Sambusiti, C., Monlau, F., Barakat, A., 2016. Bioethanol fermentation as alternative valorization route of agricultural digestate according to a biorefinery approach. Bioresour. Technol. 212, 289–295. https://doi.org/10.1016/j.biortech.2016.04.056 |
| Schievano, A., D’Imporzano, G., Salati, S., Adani, F., 2011. On-field study of anaerobic digestion full-scale plants (Part I): An on-field methodology to determine mass, carbon and nutrients balance. Bioresour. Technol. 102, 7737–7744. https://doi.org/10.1016/j.biortech.2011.06.006 |
| Seppälä, M., Pyykkönen, V., Väisänen, A., Rintala, J., 2013. Biomethane production from maize and liquid cow manure – Effect of share of maize, post-methanation potential and digestate characteristics. Fuel 107, 209–216. https://doi.org/10.1016/j.fuel.2012.12.069 |
| Somers, M.H., Jimenez, J., Azman, S., Steyer, J.-P., Baeyens, J., Appels, L., 2021. Ultrasonication affects the bio-accessibility of primary dairy cow manure digestate for secondary post-digestion. Fuel 291, 120140. https://doi.org/10.1016/j.fuel.2021.120140 |
| Stefaniuk, M., Bartmiński, P., Różyło, K., Dębicki, R., Oleszczuk, P., 2015. Ecotoxicological assessment of residues from different biogas production plants used as fertilizer for soil. J. Hazard. Mater. 298, 195–202. https://doi.org/10.1016/j.jhazmat.2015.05.026 |
| Tambone, F., Scaglia, B., D’Imporzano, G., Schievano, A., Orzi, V., Salati, S., Adani, F., 2010. Assessing amendment and fertilizing properties of digestates from anaerobic digestion through a comparative study with digested sludge and compost. Chemosphere 81, 577–583. https://doi.org/10.1016/j.chemosphere.2010.08.034 |
| Tampio, E., 2016. Utilization of Food Waste via Anaerobic Digestion: From Feedstock to Biogas and Fertilizers. Tempere University. |
| Teglia, C., Tremier, A., Martel, J.-L., 2011. Characterization of Solid Digestates: Part 2, Assessment of the Quality and Suitability for Composting of Six Digested Products. Waste and Biomass Valorization 2, 113–126. https://doi.org/10.1007/s12649-010-9059-x |
| Torrisi, B., Allegra, M., Amenta, M., Gentile, F., Rapisarda, P., Fabroni, S., Ferlito, F., 2021. Physico-chemical and multielemental traits of anaerobic digestate from Mediterranean agro-industrial wastes and assessment as fertiliser for citrus nurseries. Waste Manag. 131, 201–213. https://doi.org/10.1016/j.wasman.2021.06.007 |
| Walsh, J.J., Jones, D.L., Edwards‐Jones, G., Williams, A.P., 2012. Replacing inorganic fertilizer with anaerobic digestate may maintain agricultural productivity at less environmental cost. J. Plant Nutr. Soil Sci. 175, 840–845. https://doi.org/10.1002/jpln.201200214 |


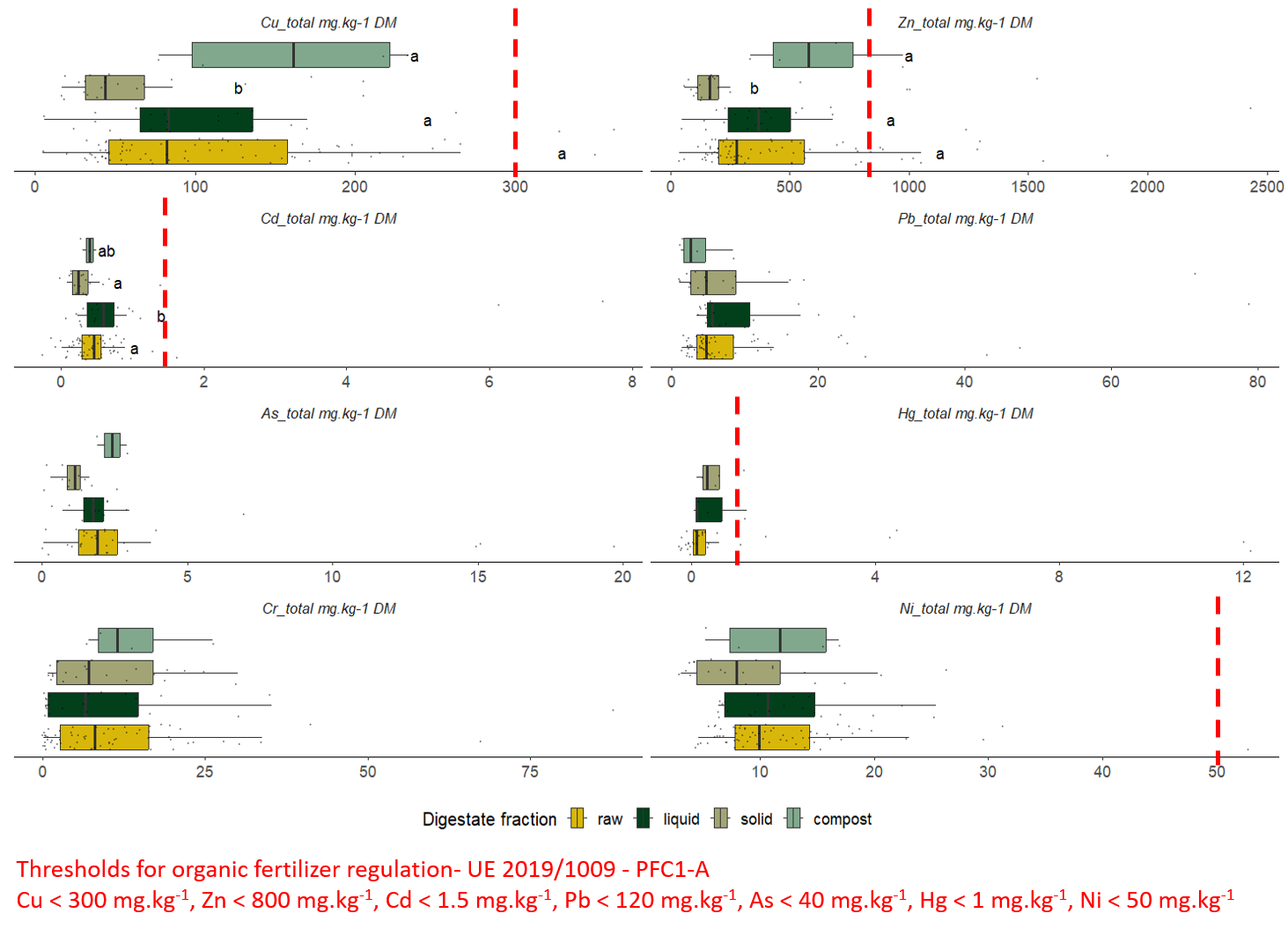


Figure S1 : Boxplots obtained from “Trace Metals Elements composition” of the collected digestates dataset and comparison with European Regulation UE 2019/1009.

Different letters indicate when significant differences appeared between groups based on Tukey's post-hoc test for ANOVA or Dunn's test for Kruskal-Wallis (p < 0.05). (Cu: copper, Zn: zinc, Cd: cadmium, Pb: lead, As: arsenic, Hg: mercury, Cr: chromium, Ni: nickel)
